# IPFMC: an iterative pathway fusion approach for enhanced multi-omics clustering in cancer research

**DOI:** 10.1093/bib/bbae541

**Published:** 2024-10-27

**Authors:** Haoyang Zhang, Sha Liu, Bingxin Li, Xionghui Zhou

**Affiliations:** Hubei Key Laboratory of Agricultural Bioinformatics, College of Informatics, Huazhong Agricultural University, No. 1 Shizishan Street, Hongshan District, Wuhan 430070, People’s Republic of China; Hubei Key Laboratory of Agricultural Bioinformatics, College of Informatics, Huazhong Agricultural University, No. 1 Shizishan Street, Hongshan District, Wuhan 430070, People’s Republic of China; Hubei Key Laboratory of Agricultural Bioinformatics, College of Informatics, Huazhong Agricultural University, No. 1 Shizishan Street, Hongshan District, Wuhan 430070, People’s Republic of China; Hubei Key Laboratory of Agricultural Bioinformatics, College of Informatics, Huazhong Agricultural University, No. 1 Shizishan Street, Hongshan District, Wuhan 430070, People’s Republic of China; Key Laboratory of Smart Farming for Agricultural Animals, Ministry of Agriculture and Rural Affairs, No. 1 Shizishan Street, Hongshan District, Wuhan 430070, People’s Republic of China

**Keywords:** data fusion, cancer subtypes, multi-omics, pathway, clustering

## Abstract

Using multi-omics data for clustering (cancer subtyping) is crucial for precision medicine research. Despite numerous methods having been proposed, current approaches either do not perform satisfactorily or lack biological interpretability, limiting the practical application of these methods. Based on the biological hypothesis that patients with the same subtype may exhibit similar dysregulated pathways, we developed an Iterative Pathway Fusion approach for enhanced Multi-omics Clustering (IPFMC), a novel multi-omics clustering method involving two data fusion stages. In the first stage, omics data are partitioned at each layer using pathway information, with crucial pathways iteratively selected to represent samples. Ultimately, the representation information from multiple pathways is integrated. In the second stage, similarity network fusion was applied to integrate the representation information from multiple omics. Comparative experiments with nine cancer datasets from The Cancer Genome Atlas (TCGA), involving systematic comparisons with 10 representative methods, reveal that IPFMC outperforms these methods. Additionally, the biological pathways and genes identified by our approach hold biological significance, affirming not only its excellent clustering performance but also its biological interpretability.

## Introduction

Cancer is a complex and highly heterogeneous disease where even tumors of the same type may exhibit substantial differences [1], impacting clinical presentation, molecular characteristics, and drug response. Personalized medicine [2] is crucial for improving the prognosis of cancer patients. Tumor subtyping is a pivotal aspect of precision medicine. Traditional subtyping, reliant on tissue or cell type origin, lacks precision due to its dependence on macroscopic features, underscoring the necessity for molecular-based subtyping. High-throughput sequencing technologies facilitate the accessible acquisition of large-scale multi-omics data [3], enabling precise subtyping based on molecular characteristics. Considering that different omics data can reflect various aspects of the cancer development process, numerous methods have been developed to integrate diverse omics data for clustering [4]. These multi-omics integration clustering methods can be categorized into three main approaches based on the stage of integration: early integration, late integration, and intermediate integration [3]. Early integration methods directly consolidate diverse omics data and subsequently employ clustering algorithms. LRACluster [5] exemplifies such an approach, utilizing an integrated probabilistic model based on low-rank approximation to discern the common principal subspace of various omics data types for integration. Late integration methods entail individual clustering of each data level using clustering algorithms and then amalgamating multiple clustering results. Perturbation clustering for data integration and disease subtyping (PINS) [6] serves as an illustration of this method, employing its proprietary perturbation clustering approach to integrate coclustering patterns across distinct omics. Currently, intermediate integration methods are predominant, which consider relationships between information from different levels during the data integration process, thereby achieving a more comprehensive data representation.

In midterm integration methods, a popular category is based on similarity matrices, such as similarity network fusion (SNF) [7], NEighborhood based Multi-Omics clustering (NEMO) [8], and MultiNMF [9]. These methods calculate sample similarity matrices for each omics data and employ techniques like non-negative matrix factorization (NMF) to merge different omics' similarity matrices into a unified matrix for clustering. The statistical approaches, such as iClusterBayes [10], utilize Bayesian latent variable regression models to project different omics into a common low-dimensional integrated space. In recent years, deep learning algorithms have been applied to multiple biomedical fields, such as Heterogeneous Graph Transformer for Drug Repurposing (HGTDR) [11] for drug repurposing and CFSSynergy [12] for drug synergy prediction. The same trend can be observed in the field of multi-omics cancer subtyping. For instance, Subtype-GAN [13] connects multiple independent omics layers to a shared layer, leveraging hidden factors for consensus clustering. Another published method, Multi-Reconstruction Graph Convolutional Network (MRGCN) [14], integrates graph convolutional networks to compute embedding representations for each omics dataset and unify them through a joint optimization framework, effectively addressing the challenge of multi-omics data integration.

While the aforementioned methods have made some progress, they often suffer from a lack of biological interpretability, which limits their broader application. In response, some researchers have incorporated prior knowledge, such as biological pathway information, into clustering frameworks to enhance biological interpretability. For instance, PathME [15] divides omics data by pathway, scores each pathway using an autoencoder, and employs NMF and biclustering to classify patients into different subtypes. Pathway-based MultiOmic Graph Kernel clustering (PAMOGK) [16] represents patients as vertex-labeled undirected graphs based on pathways, utilizing kernel methods to capture the topological similarity for interpretable clustering. However, these methods are challenging to apply to other datasets due to their complex structure and dependencies on specific data formats, making them less user-friendly and lacking systematic performance evaluation across multiple cancers. To address these challenges, we propose an Iterative Pathway Fusion approach for enhanced Multi-omics Clustering (IPFMC), a structurally simple and biologically interpretable multi-omics clustering framework. Based on the biological hypothesis that patients with the same subtype may exhibit similar dysregulated pathways, while patients with different subtypes may have distinct dysregulated pathways, IPFMC employs a two-stage fusion method to integrate multiple omics data. In the first stage, for each omics data, it divides the omics data into different pathway subsets and computes the sample similarity networks for each pathway subset. Then IPFMC integrates all the similarity matrices, resulting in a common representation of all pathways. Subsequently, IPFMC iteratively filters out the important pathways and calculates the common representation of the remaining pathways for each layer of omics data and ranks the pathways according to their consistency with the common representation to provide biological interpretability. In the second stage, IPFMC integrates the representation of all omics data using SNF [7] to obtain the final similarity between patient samples and uses spectral clustering [17] to divide the cancer patients into different subtypes. Compared to other methods based on prior knowledge, the advantage of IPFMC is its high performance and simple structure. The IPFMC implementation code and evaluation code are available at https://github.com/BioLemon/IPFMC.

To evaluate IPFMC, we systematically compared it against 10 representative methods using multi-omics datasets [messenger RNA (mRNA) expression data, microRNA (miRNA) expression data, DNA methylation data, and copy number variation (CNV) data] from nine cancer types with the largest samples in TCGA [18]. The results indicate that our approach excels in clustering cancer subtypes and demonstrates superior performance in survival analysis and gold-standard dataset evaluation. Furthermore, a case study on a lung cancer dataset illustrates the biological interpretability of our method by identifying pathways and genes associated with lung cancer occurrence.

## Materials and Methods

In this section, the datasets used in this study, the steps of IPFMC, and the evaluation methods will be detailed.

### Datasets and preprocessing

In a previous work [4], four types of omics data (mRNA expression, microRNA expression, DNA methylation, and CNV) across nine cancers [adrenocortical carcinoma (ACC), breast-invasive carcinoma (BRCA), colon adenocarcinoma (COAD), kidney renal papillary cell carcinoma (KIRP), kidney renal clear cell carcinoma (KIRC), liver hepatocellular carcinoma (LIHC), lung adenocarcinoma (LUAD), lung squamous cell carcinoma (LUSC), and thymoma (THYM)] from TCGA were selected to evaluate the performance of multi-omics data clustering methods. The true labels of two data sets (BRCA and COAD) were also obtained from this work. In our work, we employ the same datasets and preprocessing method to assess the effectiveness of our proposed approach.

The gene sets of all the pathways were downloaded from MSigDB (https://www.gsea-msigdb.org/gsea/msigdb/human/collections.jsp#C2), and canonical pathways from the curated gene sets (C2) were used in this work (only pathways with 10–200 genes were selected to avoid pathways whose gene sets were too generic or too specific). The miRNA target information was collected from miRTarBase (Release 9.0 beta) [19]. To establish the relationship between miRNA expression data and biological pathways, we categorized miRNAs whose targets were significantly enriched in genes associated with specific pathways into their corresponding pathways. We employed hypergeometric and distribution tests to assess the enrichment of miRNAs (with their target gene set denoted as A) in specific pathways (with the pathway gene set denoted as B). The calculation is as follows:


(1) \begin{equation*} P\left(X=k\right)=\frac{\left(\genfrac{}{}{0pt}{}{M}{k}\right)\left(\genfrac{}{}{0pt}{}{N-M}{n-k}\right)}{\left(\genfrac{}{}{0pt}{}{N}{n}\right)} \end{equation*}

where *X* is the intersection size, representing the common genes between Set A and Set B, *N* is the population size of the hypergeometric distribution test, *M* is the number of set A, and *n* is the number of Set B. In this study, to avoid an excessive number of miRNAs enriching in the same pathway, we considered miRNA–pathway relationships with *P*-values <.005 as significant associations.

### The details of IPFMC

As mentioned earlier, IPFMC integrates multiple similarity matrices to construct a common representation of multi-omics pathways and iteratively selects important pathways for similarity network fusion and cancer subtype identification via spectral clustering [17]. IPFMC employs a two-stage fusion strategy to integrate multiple omics datasets for a comprehensive representation of each sample. In the first fusion stage, IPFMC utilizes prior knowledge (i.e. pathway gene sets) to partition each omics dataset into multiple subsets. It calculates sample similarity matrices based on each gene set, iteratively selects consistent pathways, and ultimately integrates the similarity matrices corresponding to these consistent pathways to obtain sample similarity matrices for each omics dataset. In the second fusion stage, we use SNF [7] to integrate the similarity matrices of different omics datasets, resulting in the final sample similarity matrix. Spectral clustering [17] is then applied for sample clustering.

#### Data input requirements for IPFMC

IPFMC accepts a multi-omics dataset $X=\left\{{X}^{(1)},{X}^{(2)},\dots, {X}^{(L)}\right\},{X}^{(l)}\in{R}^{m_l\ast n}$as input, where m*_l_* represents the number of features of *l*-th omics data, *n* represents the number of samples, and *L* represents the number of omics. The column index of each omics data in the dataset should be the patient’s ID, and the row index should be the feature name (such as gene name). By default, IPFMC uses pathways as the prior knowledge.

#### Fusion method of the first stage

At this stage, we select representative pathway information for each omics dataset to characterize the samples and integrate them to obtain a unified sample similarity matrix. Subsequently, we employ two strategies for information fusion. Experimental results indicate that clustering samples using Strategy 1 yields better survival differences, while Strategy 2 produces results that are more consistent with the existing well-established subtypes. Both strategies are parameterized in our toolkit, allowing users to choose based on their specific requirements. The detailed procedures for these two strategies are outlined below, using the *l*-th omics data as an example.

##### Strategy 1: *K*-means discretization strategy

The first strategy was inspired by SC3 [20]. Assuming a given pathway consists of *w* features (genes or miRNA), the Euclidean distance is employed to assess the relationship between any two samples within a specific omics dataset. The calculation of the Euclidean distance is as follows:


(2) \begin{equation*} d\left(p,q\right)=\sqrt{{\left({p}_1-{q}_1\right)}^2+{\left({p}_2-{q}_2\right)}^2+\dots +{\left({p}_w-{q}_w\right)}^2} \end{equation*}

(3) \begin{equation*} {D}_k^{(l)}\left(i,j\right)=d\left({N}_i,{N}_j\right) \end{equation*}

where *p* and *q* represent the feature vectors of any two samples, $d\left(p,q\right)$ is the Euclidean distance between the *p* vector and the *q* vector, ${D}_k^{(l)}\left(i,j\right)$ is the value of the *i*-th row and *j*-th column of the Euclidean distance matrix of the *k*-th pathway, and $d\left({N}_i,{N}_j\right)$ is the distance between the *i*-th sample and the *j*-th sample.

Next, IPFMC employs principal component analysis to process the Euclidean distance matrix of each pathway, a strategy previously utilized in SC3 [20]. We retain the top *d* principal components from each Euclidean distance matrix, forming an *n*-by-*d* matrix. This matrix encapsulates the predominant information of the current pathway within the specific omics dataset. Here, we set d to be 5% of the sample size (i.e. *n*).

Then, IPFMC performs *k*-means++ [21] clustering on the *n*-by-*d* matrix and obtains the cluster label vector ${v}_k$ of each sample. After that, IPFMC uses the cluster-based similarity partitioning algorithm [22] to calculate the binary similarity matrix ${S}_k^{(l)}\in{R}^{n\times n}$ of *k*-th pathway cluster labels. If two samples belong to the same cluster in the cluster label vector, the corresponding position of the binary similarity matrix is set to 1, otherwise 0, that is:


(4) \begin{equation*} {S}_k^{(l)}\left(i,j\right)=\left\{\begin{array}{@{}ll}1,&{v}_k(i)={v}_k(j)\\{}0,&{v}_ki\ne{v}_k(j)\end{array}\right. \end{equation*}

For each pathway subset of each omics, IPFMC obtains the corresponding pathway *K*-means clusters and binary similarity matrix according to the above strategy and obtains the cluster label vector set ${V}^{(l)}=\{{v}_1^{(l)},{v}_2^{(l)},\dots, {v}_K^{(l)}\}$ and the binary similarity matrix set ${B}^{(l)}=\{{S}_1^{(l)},{S}_2^{(l)},\dots, {S}_K^{(l)}\}$.

Next, IPFMC calculates the mean of all the binary similarity matrices of the pathways to obtain the integrated representation${T}^{(l)}$:


(5) \begin{equation*} {T}^{(l)}=\frac{S_1^{(l)}+{S}_2^{(l)}+\dots +{S}_K^{(l)}}{\mathrm{K}} \end{equation*}

IPFMC performs spectral clustering [17] on ${T}^{(l)}$ to obtain the cluster label vector of each sample on ${T}^{(l)}$, denoted as $\overset{\sim }{v}$:


(6) \begin{equation*} {T}^{(l)}\overset{Spectral\ Clustering}{\Rightarrow}\overset{\sim }{v} \end{equation*}

Next, the Adjusted Rand Index (ARI) is employed to assess the consistency between the clustering label (${V}^{(l)}$) of each pathway and the fused label vector ($\overset{\sim }{v}$) mentioned above. Only those pathways with high ARI values are retained. Subsequently, based on the retained pathways' clustering label vectors, the fused label is recalculated, and high-consistency pathways are filtered. Through multiple iterations of fusion and filtering (the number of iterations is set as 6 in this work), a final sample representation (${F}^{(l)}\in{R}^{n\times n}$) is obtained for the current omics dataset, along with the list of retained pathways (${H}^{(l)}$). The former is used for the second stage of information fusion, while the latter can be employed for biological functional interpretation.

##### Strategy 2: Average representation strategy

First, for each omics dataset, the Euclidean distance matrix is calculated using the features within each pathway (consistent with Strategy 1) and then converted to the intersample similarity matrix using the following formula:


(7) \begin{equation*} {R}_k^{(l)}={e}^{-\frac{D_k^{(l)}}{\mathit{\max}\left({D}_k^{(l)}\right)}} \end{equation*}

where ${D}_k^{(l)}$ represents the Euclidean distance matrix of the *k*-th pathway and ${R}_k^{(l)}$ represents the transformed similarity matrix of the k-th pathway. Subsequently, the sample representation for the current omics dataset (initial common representation) ${T}^{(l)}$is obtained by averaging the similarity matrices from all pathways directly:


(8) \begin{equation*} {T}^{(l)}=\frac{R_1^{(l)}+{R}_2^{(l)}+\dots +{R}_K^{(l)}}{K} \end{equation*}

Next, the Manhattan distance is computed between the representation (similarity matrix) of each pathway and the common representation (average matrix). Iterative filtering is then performed based on Manhattan distance, retaining only those pathways with consistency (i.e. pathways with relatively smaller Manhattan distances to the common matrix). The updated sample representation is obtained by averaging the similarity matrices of the retained pathways. This process is iterated multiple times (consistent with Strategy 1, six iterations), resulting in a common representation matrix composed of retained consistent pathways, denoting as ${F}^{(l)}\in{R}^{n\times n}$. The formula for calculating the Manhattan distance is as follows:


(9) \begin{equation*} d\left(p,c\right)={\sum}_{i=1}^{n^2}\left|{p}_{\mathrm{i}}-{c}_{\mathrm{i}}\right| \end{equation*}

where *p* is the vector obtained by unfolding the Euclidean distance matrix of the current pathway, and *c* is the vector obtained by unfolding the common representation matrix.

#### Similarity network fusion integrates multi-omics representations

In the previous step, the representation of each omics data was performed based on the important pathways. Then, IPFMC uses the SNF [7] to integrate the similarity representations of multiple omics. Let the set of common representation matrices of all omics data be $A=\left\{{F}^{(1)},{F}^{(2)},\dots, {F}^{(L)}\right\}$ and ${F}^{(l)}\left(i,j\right)$ be the similarity between samples $i$ and $j$ of *l*-th omics; let ${N}_i^{(l)}$ be the neighbor set of sample $i$ of *l*-th omics, SNF first calculates the normalized weight matrix ${P}^{(l)}$:


(10) \begin{equation*} {P}^{(l)}\left(i,j\right)=\left\{\begin{array}{@{}ll}\frac{F^{(l)}\left(i,j\right)}{\sum_{k\ne i}{F}^{(l)}\left(i,k\right)}, & j\ne i\\{}1/2, & j=i\end{array}\right. \end{equation*}

Then, SNF calculates a local affinity matrix between samples by the following formula:


(11) \begin{equation*} {O}^{(l)}\left(i,j\right)=\left\{\begin{array}{@{}ll}\frac{W\left(i,j\right)}{\sum_{k\in{N}_i^{(l)}}W\left(i,k\right)}, & j\in{N}_i\\{}0, & otherwise\end{array}\right. \end{equation*}

After obtaining the normalized weight matrix and the local affinity matrix between samples, SNF iteratively fuses the normalized weight matrix and the local affinity matrix by a message-passing process:


(12) \begin{equation*} {P}^{(l)}={O}^{(l)}\times \left(\frac{\sum_{k\ne l}{P}^{(k)}}{m-1}\right)\times{\left({O}^{(l)}\right)}^T,l=1,2,\dots, L \end{equation*}

After *t* iterations, each omics data will obtain a state matrix, and then, the state matrix is averaged to obtain the final data representation ${P}^{(c)}$:


(13) \begin{equation*} {P}^{(c)}=\frac{\sum_{i=1}^L{P}_t^{(i)}}{L} \end{equation*}

#### Clustering based on the fused multi-omics representation

Spectral clustering [17] was applied to cluster the samples into different cancer subtypes based on the multi-omics representation of the samples. IPFMC determines the recommended number of clusters by selecting the one that yielded the highest silhouette coefficient [23] when needed.

### Performance evaluation

This study employed a similar evaluation methodology as in previous work [4]. We utilized four types of omics data, with each method performing clustering on any combination of two, three, and all four omics data. The number of clusters ranged from 2 to 8, and we systematically compared the comprehensive performance of each method based on different clustering results. Performance comparisons included survival analysis results for clustering across all nine cancer types and evaluations against gold-standard clustering results on two benchmark datasets. For survival analysis, log-rank tests [24] were used to assess whether there were significant differences in the survival information of patients with different subtypes. We used the number of significant log-rank *P*-values and log-rank *P*-value distribution to evaluate methods’ performance on survival analysis (for detailed calculation of the number of significant log-rank *P*-values and log-rank *P*-value distribution, see Supplementary Note 1). As for benchmark datasets, ARI, normalized mutual information (NMI), Precision, and F-score were used to evaluate the cluster results with true labels. For the details of all the indices, please refer to Supplementary Note 2.

## Results

In the Results section, we provide a concise overview of the IPFMC. Subsequently, we present comparative experiments with other methods, focusing on survival analysis and gold-standard dataset evaluation. To showcase the biological interpretation capabilities of our method, we present a case study using the lung cancer dataset. Finally, ablation experiments are conducted to highlight the effectiveness of each step in our method.

### Framework of IPFMC

**Figure 1:**
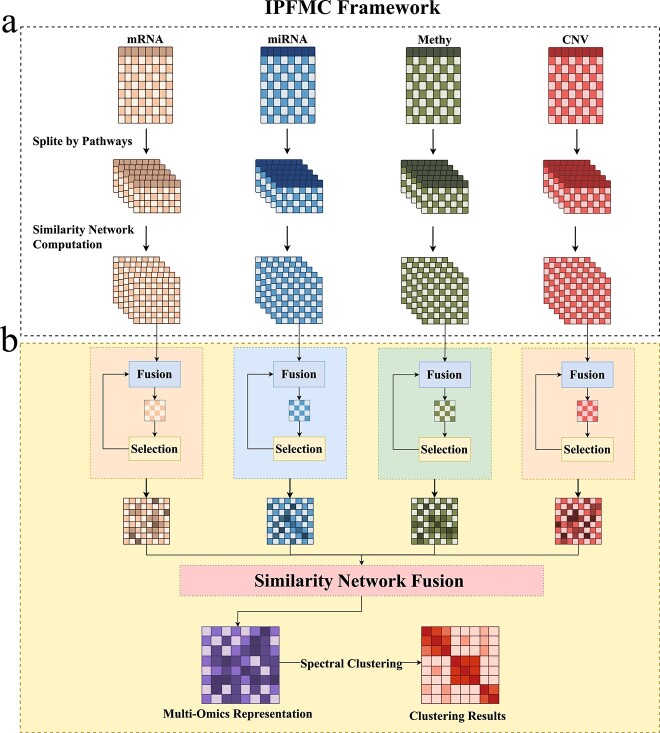
The framework of IPFMC. (a) Construction of pathway similarity matrix. After obtaining the omics data, IPFMC first extracts them into different pathway subsets according to the pathway information and calculates the similarity network for each pathway. (b) Two-stage fusion. IPFMC performs a consistency-based iterative pathway screening to select the important similarity networks and integrate them from the representation for each omics data and then fuses the omics representations using the SNF strategy to obtain the multi-omics representation. At last, spectral clustering was performed to divide the patients into different cancer subtypes.

Different dysregulated pathways may play distinct roles in various subtypes of cancer [25]. Building on this observation, IPFMC integrates pathway information into cancer subtyping based on multi-omics data using a two-stage fusion strategy. In the first stage, for each omics data, IPFMC partitions the data into different subsets based on pathway information. It calculates the similarity network between samples using data from each subset, iteratively selects important pathways to represent samples, and fuses information from the similarity networks of the important pathways to obtain a common similarity network for each omics data. In the second stage, similar to the SNF method, it integrates the similarity networks of multiple omics data to obtain a final sample representation matrix. Finally, spectral clustering [17] is applied to classify samples into different subtypes. The main framework of IPFMC is illustrated in Fig. 1 (detailed methods are described in the Materials and Methods section).

### Comparison with state-of-art methods

In a previous review article, Duan *et al*. systematically evaluated 10 representative multi-omics clustering methods [4], including survival analysis and benchmark data assessment. In this study, we adopted a similar assessment strategy with the 10 methods. These methods include an early integration method (LRACluster [5]), a late integration method (PINS [6]), and eight intermediate integration methods: five network-based methods (SNF [7], NEMO [8], CIMLR [26], MultiNMF [9], and PFA [27]), two statistics-based methods (moCluster [28] and iClusterBayes [10]), and one deep learning–based method (Subtype-GAN [13]). We also attempted to compare with pathway-based methods; however, these methods were challenging to implement, and their evaluations were typically limited to a small number of datasets. Fortunately, the paper of PAMOGK [16] provided guidelines on how to apply their method to the KIRC cancer dataset, allowing us to compare the survival analysis results on KIRC with PAMOGK.

#### Survival analysis

**Figure 2:**
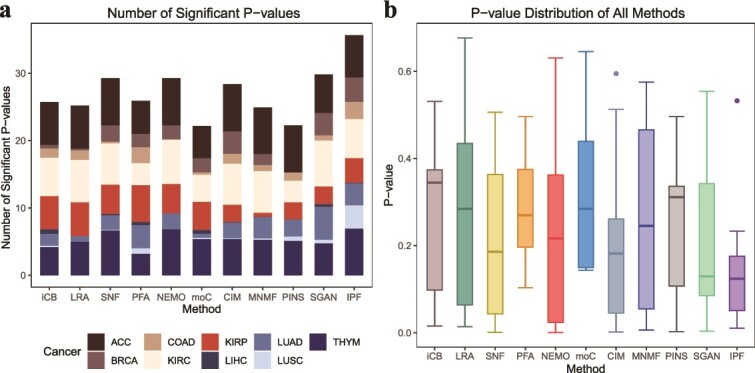
Performance of IPFMC in survival analysis. We use “iCB,” “LRA,” “moC,” “CIM,” “MNMF,” “SGAN,” and “IPF” to represent iClusterBayes, LRAcluster, moCluster, CIMLR, MultiNMF, Subtype-GAN, and IPFMC, respectively. (a) Number of significant log-rank *P*-values for each method. (b) Average of all cancers’ log-rank *P*-values’ distribution for each method.

In the survival analysis, we conducted a large-scale comprehensive comparison. Each cancer type comes with four kinds of omics data, leading to 11 possible combinations of these data types. For every combination, we tested the number of clusters ranging from 2 to 8. This setup resulted in 77 separate clustering results for each cancer type. When applied to all nine cancer datasets, each method was evaluated across a total of 693 distinct clustering results. For each clustering outcome, survival analyses were conducted to determine whether patients in different clusters exhibited significant differences in survival risk, utilizing the log-rank test. The results of the survival analyses for the 10 methods are presented in Fig. 2, with detailed results available in Supplementary Table 1.

Figure 2a displays the frequency of significant survival analysis results for each method. The figure clearly demonstrates that our method yields a notably higher number of clustering results showing prognosis differences across various clusters compared to alternative methods. Following are Subtype-GAN [13], SNF [7], NEMO [8], and other methods with relatively inferior performance. Figure 2b shows the survival risk differences among patients in different groups in our clustering results are noticeably more significant. Following are Subtype-GAN [13] and other methods, which are relatively less pronounced. To statistically validate these findings, we conducted Wilcoxon tests to compare IPFMC with other methods in terms of the number of significant *P*-values and *P*-value distribution. The results show that our method outperforms others, with statistically significant or marginally significant differences in almost all comparisons (Supplementary Table 2).

In the comparison with another pathway-based clustering method, PAMOGK [16] (Supplementary Fig. 1), our method also outperformed PAMOGK. Considering that our method has been compared with other mainstream methods on large-scale datasets, we can conclude that our method exhibits superior performance in survival analysis evaluation.

#### Gold-standard dataset evaluation

Among the nine cancer datasets, BRCA and COAD already have well-established subtypes [29]. Here, we used these two datasets as gold-standard datasets to compare our method with 10 other methods. We utilized ARI, NMI, Precision, and F-score to evaluate the performance of each method (note that since PINS does not yield subtyping outcomes for a predetermined number of clusters (*k*), its Precision and F-score could not be computed). Initially, we conducted this evaluation using Strategy 1 of IPFMC; however, we noticed a substantial discrepancy between IPFMC's output and the benchmark labels. Consequently, we carried out survival analysis on the cluster labels produced by our methodologies alongside the benchmark labels. The log-rank test results revealed that our method significantly outperformed the well-established subtypes for BRCA and marginally surpassed those for COAD (refer to Supplementary Fig. 2), suggesting that our approach might generate subtypes that more effectively differentiate survival outcomes compared to the established subtypes of BRCA and COAD. Despite this, considering the generally favorable clinical relevance of these well-established cancer subtypes, we devised Strategy 2 of IPFMC—outlined in the Materials and Methods section—to yield outcomes that align more closely with these recognized subtypes. The outcomes of this adapted strategy are presented in Table 1. Each value in the table is calculated by averaging the results from 11 omics combinations for the respective indicator, using the number of clusters (*K*) as recommended by each method for both the ARI and NMI for both cancer types. For Precision and F-score, *K* was set to 5 for BRCA and 4 for COAD (for detailed results, see Supplementary Tables 3–5).

**Table 1:** Performance of each method on the two gold-standard datasets.

Method	BRCA				COAD			
	ARI	NMI	Precision	F-score	ARI	NMI	Precision	F-score
iClusterBayes	0.163	0.233	0.447	0.491	0.145	0.207	0.462	0.525
LRAcluster	0.214	0.292	0.461	0.494	0.133	0.209	0.463	0.526
SNF	0.295	**0.389**	**0.576**	**0.614**	0.225	0.279	0.500	0.579
PFA	0.111	0.112	0.486	0.432	0.005	0.047	**0.539**	0.502
NEMO	0.270	0.365	0.556	0.600	0.228	0.273	0.528	**0.585**
moCluster	0.125	0.127	0.495	0.463	0.059	0.107	0.464	0.472
CIMLR	0.227	0.316	0.536	0.569	0.133	0.163	0.448	0.511
MultiNMF	0.227	0.298	0.478	0.529	0.036	0.046	0.373	0.430
Subtype-GAN	0.225	0.307	0.489	0.539	0.144	0.187	0.453	0.518
PINS	0.208	0.288	–	–	0.132	0.204	–	–
IPFMC	**0.299**	0.341	0.549	0.582	**0.333**	**0.293**	0.506	0.579

The results showed that our method achieved the highest ARI for BRCA, as well as the highest ARI and NMI for COAD. Additionally, SNF attained the best NMI, Precision, and F-score on the BRCA dataset. Overall, our method and SNF performed comparably on these two benchmark datasets, achieving the best performance, followed by NEMO. This indicates that our method had a high consistency with the well-established subtypes when using the recommended *K*, confirming that our method had a high consistency with the previously recognized cancer molecular subtyping results.

### Biology interpretation—a case study on lung cancer

In the data integration of stage I, IPFMC iteratively selects important pathways to represent samples, so pathways with higher rankings may be closely related to cancer subtyping. Here, we perform an analysis on the LUAD dataset to showcase the top 10 pathways for each omics data, aiming to examine whether our method can genuinely identify pathways of significant biological importance.

#### Case study on important pathways

**Figure 3:**
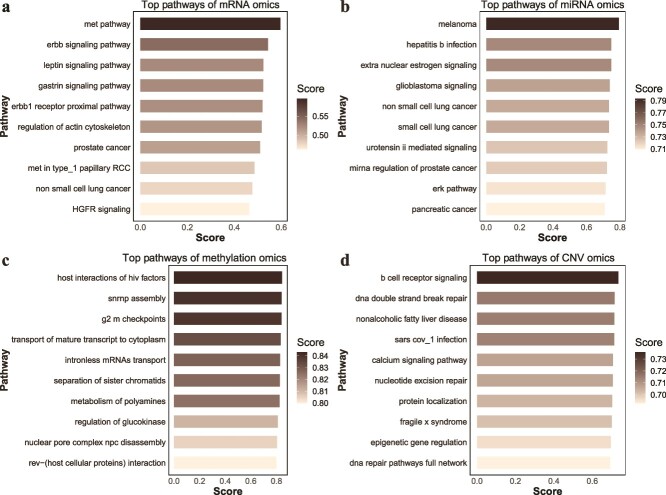
The top 10 pathways and their ARI scores of four types of omics data [(a) mRNA, (b) miRNA, (c) methylation, and (d) CNV] obtained by IPFMC (limited by the size of the image display, some pathway names were abbreviated).

In LUAD, the top 10 pathways of each omics data are shown in Fig. 3. For detailed information on all the selected pathways in each omics dataset, please refer to Supplementary Tables 6–9.

**Figure 4:**
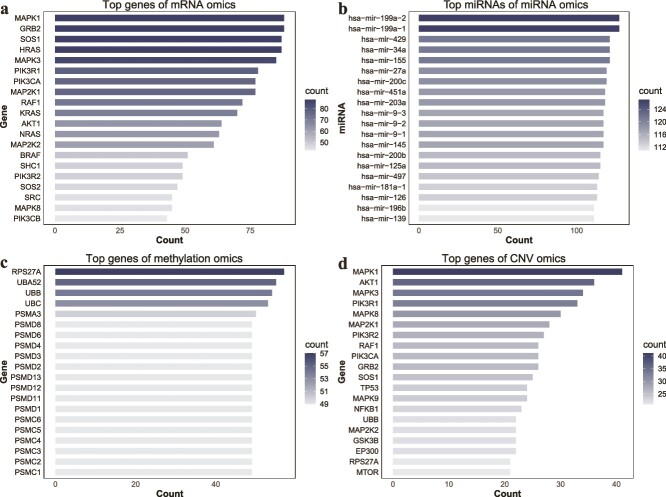
The top 20 gene/miRNA and their number of occurrences in four types of omics data [(a) mRNA, (b) miRNA, (c) methylation, and (d) CNV] obtained by IPFMC.

Overall, each omics type can capture numerous cancer-related biological pathways. In the mRNA data, the top-ranked pathway is the "met pathway," identified as a significant oncogenic driver in a lung cancer subgroup, affirming its close association with lung cancer [30]. Additionally, the Hepatocyte Growth Factor Receptor (HGFR) pathway has been established as crucial in nonsmall cell lung cancer [31]. Other well-known pathways related to lung cancer, such as the erbb signaling pathway, erbb1 receptor proximal pathway, and nonsmall cell lung cancer, also rank in the top 10. This underscores that our pathway iterative selection process effectively identifies pathways highly relevant to lung cancer in mRNA data processing. In the miRNA expression data, our method not only identified biological pathways related to lung cancer, such as non-small cell lung cancer ranking fifth and small cell lung cancer ranking sixth, but also selected pathways associated with other cancers. For instance, melanoma ranked first, glioblastoma signaling ranked fourth, and pancreatic cancer ranked 10th.

Meanwhile, compared to the previous two types of omics data, the top pathways in DNA methylation and CNV data were not as enriched in direct cancer-related pathways but rather in other cancer hallmark-related pathways. The main pathways in DNA methylation data were related to cell proliferation, including the G2-M checkpoints pathway ranked third, the separation of sister chromatids pathway ranked sixth, and the nuclear pore complex disassembly pathway ranked ninth. In CNV data, the top 10 pathways were mainly associated with nucleic acid repair, such as the DNA double-strand break repair pathway ranked second, the nucleotide excision repair pathway ranked sixth, and the DNA repair pathways full network pathway ranked 10th.

In summary, these findings affirm the robust capability of our method in enriching cancer-related pathways. We observed that pathways enriched by distinct omics modalities appear to capture various facets related to cancer. In the presented lung cancer results, mRNA and miRNA-omics demonstrated a tendency to enrich pathways directly associated with cancer, while methylation-omics and CNV-omics each enriched distinct biological processes related to cell differentiation and cell cycle–associated pathways. This observation may indicate that our method extracts information from different layers in various omics data, ensuring a comprehensive sample representation during the multi-omics integration process and thereby ensuring excellent clustering outcomes.

#### Case study on top genes

We further examined whether our method could identify genes associated with cancer. In each omics data, genes were ranked based on their occurrence frequency in the selected pathways, and the top 20 genes with the highest frequencies are shown in Fig. 4. From the figure, it can be observed that in both mRNA expression and CNV data, MAPK1 ranked first. Other genes from the MAPK1 gene family, such as MAPK2, MAPK3, and MAPK8, also ranked prominently. Additionally, RAF1 appeared in both omics datasets. Notably, the MAPK pathway and the RAF pathway are well-established pathways closely linked to cancer [32]. Furthermore, the KRAS gene ranked 10th in mRNA data, and it is a gene closely associated with lung cancer, with KRAS mutations present in 35% of LUAD cancer [33]. The TP53 gene ranked 12th in CNV data, and TP53 is one of the most renowned cancer-related genes [34]. The genes enriched by DNA methylation were quite different from those of mRNA and CNV omics. The gene with the most occurrence times was RPS27A, and previous studies have found that this gene regulates cell cycle, regulation, and apoptosis in LUAD cancer cells [35]. In addition, many PSMC and PSMD family genes appeared in the gene list, and previous studies have mentioned that PSMC and PSMD family genes are related to cell cycle and cytoskeleton remodeling [36, 37], which is consistent with pathway enrichment of DNA methylation data.

**Figure 5:**
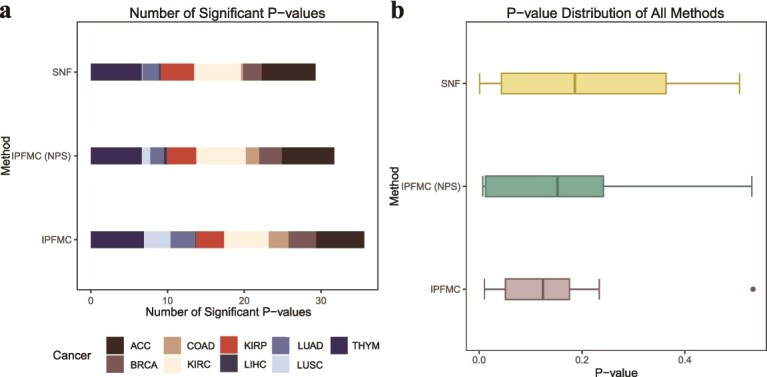
Ablation experiment results. (a) The number of significant *P*-values. (b) The average *P*-value distribution. The definition is the same as in Fig. 2.

For the miRNA expression data, the top-ranked hsa-mir-199a contained the GRP78 gene in its target genes, and previous studies have confirmed that the overexpression of hsa-mir-199a-5p was related to the downregulation of GRP78 gene expression, and, in nonsmall cell lung cancer, GRP78 gene expression was significantly upregulated [38], confirming that hsa-mir-199a was an miRNA closely related to lung cancer. For additional cancer-associated miRNAs and their corresponding supporting literature, please refer to Supplementary Table 10.

### Ablation experiment

Our method employs a two-stage fusion strategy, with the second stage similar to SNF [7]. In comparison to SNF [7], our method offers two primary contributions. The first involves the use of pathway information in the first fusion phase. The second contribution lies in employing an iterative process to select crucial pathways for sample representation in the first stage. In the Ablation Experiment section, we compare the clustering performance of SNF [7] with a strategy that does not iteratively select pathways in the first fusion stage [i.e. using all pathways for data fusion representation, denoted as IPFMC - no pathway selection (NPS)] and our method (IPFMC). As survival analysis is a commonly used metric for different clustering methods, and it is the primary comparative method in this study, we assess the performance of the three methods through survival analysis in the ablation experiments.

As depicted in Fig. 5, both the frequency of significant results in survival analysis and the distribution of *P*-values in survival analysis demonstrate that the strategy of using all pathways in the first fusion stage surpasses the direct application of SNF [7]. Moreover, the best results are achieved when crucial pathways are selected for the first fusion stage, as in our method. In conclusion, the ablation studies indicate that both primary contributions of our method are essential for achieving superior clustering results.

## Discussion

Performing clustering based on multi-omics data is crucial for precision medicine research. While several multi-omics methods have been developed, they often lack biological interpretability, limiting their applicability and affecting clustering performance. Despite a few methods incorporating pathway information into clustering to provide biological interpretability [15, 16], these methods have mainly been analyzed on a limited number of cancers, lacking comprehensive evaluation. Additionally, their complexity makes them less user-friendly. In this study, we propose IPFMC, a structurally simple yet biologically interpretable multi-omics clustering method. IPFMC employs a two-stage fusion strategy, iteratively selecting important biological pathways in the first stage to divide each omics dataset into subsets, using subset data for sample representation, and integrating the representations of all subsets after iteration to obtain the sample representation for each omics. In the second stage, the well-performing SNF [7] method is used to fuse multiple omics data together. Comprehensive evaluation across multiple datasets demonstrates our method's superior clustering results. Furthermore, the biological interpretability of our method is evident in the selected pathways and important genes.

Despite the promising results, there are some issues that still need improvement. For example, in the iterative selection of important pathways for sample representation, we choose pathways with higher consistency with the common representation of all pathways. Although validation experiments show its benefits for clustering, it might not always yield the optimal pathway combination. Effectively, complementary subset combinations may perform better than directly integrating highly ranked and consistent pathways. This will be addressed in our future work. We calculated the runtime of IPFMC on a system with an Intel(R) Xeon(R) Platinum 8375C CPU @ 2.90GHz and 64GB of memory. We found that the runtime for Strategy 1 was relatively long, ranging from 311 s (for the ACC cancer dataset with 77 samples) to 6035 s (for the BRCA cancer dataset with 759 samples). In contrast, Strategy 2 exhibited a shorter runtime, ranging from 21 to 240 s. Since our method uses each pathway to represent the samples, it requires substantial computational time. Additionally, our package incorporates all available pathway data (including 2848 pathways), which further increases the runtime. We tested running our model (Strategy 1) with fewer pathways by selecting a subset of pathways with the highest median absolute deviation. The results show that pre-filtering the pathways does not significantly impact clustering performance (Supplementary Fig. 3a) while reducing the runtime substantially (Supplementary Fig. 3b). In our package, we also provide an interface that allows users to select a specific proportion of pathways to run the model (as including all pathways could offer more comprehensive functional annotation, our model defaults to using all pathways). Additionally, our method currently only uses bulk genomic data. With the widespread use of single-cell data, adapting our method to handle single-cell multi-omics data represents a significant challenge that we aim to address in our future research.

Furthermore, in addition to its effectiveness, we strive to enhance the usability of our method. We provide not only straightforward code with a user-friendly interface on GitHub but also a Python package with detailed instructions for users' ease. We hope these efforts will facilitate more researchers in utilizing our method. In summary, we have introduced IPFMC, a multi-omics clustering model that is designed to effectively differentiate biologically meaningful cancer subtypes, for molecular subtyping of cancer in biomedical research. This model excels not only in clustering performance but also in providing biological interpretability, making it accessible and useful for researchers. We believe that our method can provide valuable support for cancer research.

**Key Points**

- Based on the biological hypothesis that patients with the same subtype may exhibit similar dysregulated pathways, IPFMC incorporates pathway information into a multi-omics data integration process and proposes a two-stage fusion framework to represent cancer samples, resulting in a high-performance cancer subtyping model.
- Through systematic evaluation, IPFMC demonstrated better clustering performance compared to 10 state-of-the-art methods. The case study also showed that IPFMC could identify key biological pathways and factors involved in cancer development.
- To enhance the usability of the model, IPFMC is an easy-to-use toolkit.

## Supplementary Material

IPFMC_BIB_Supplementary_bbae541

## Data Availability

Data and codes in our experiments are released on GitHub at: https://github.com/BioLemon/IPFMC.
