# Supplementary material for: IPFMC: an iterative pathway fusion approach for enhanced multi-omics clustering in cancer research
Source: Brief Bioinform. 2024 Oct 27;25(6):bbae541. doi: 10.1093/bib/bbae541 (PMC11514061; doi:10.1093/bib/bbae541)
Supplement: IPFMC_BIB_Supplementary_bbae541 [file ipfmc_bib_supplementary_bbae541.docx]

**SUPPLEMENTARY INFORMATION FOR**

**IPFMC: An Iterative Pathway Fusion Approach for Enhanced Multi-omics Clustering in Cancer Research**

Haoyang Zhang^1#^, Sha Liu^1#^, Bingxin Li^1^, Xionghui Zhou^1,2*^

^1^Hubei Key Laboratory of Agricultural Bioinformatics, College of Informatics, Huazhong Agricultural University, Wuhan, 430070 People’s Republic of China

^2^Key Laboratory of Smart Farming for Agricultural Animals, Ministry of Agriculture and Rural Affairs, People’s Republic of China

#These authors contribute equally to the work.

^*^To whom correspondence should be addressed. (zhouxionghui@mail.hzau.edu.cn; zhouxionghui6@gmail.com)

**Supplementary Information includes:**

Supplementary Notes 1 to 2.

Supplementary Figures 1 to 3.

Supplementary Tables 1 to 12.

Supplementary References.

**Supplementary Note 1: Calculation of Survival Analysis indicators**

Number of significant log-rank p-values: Denote T as the number of cancer types (9 in this experiment), D as the number of omics combinations (11 in this experiment), and C as the number of cluster types (we set the number of clusters to 2-8 in this experiment, resulting in 7 cluster types). Denote S as the number of significant p-values (< 0.05) among C cluster results’ log-rank p-values. Then, for a given method, the Number of significant log-rank p-values (N) is defined as follows:

$$\begin{aligned} N=\sum_{t=1}^{T} \frac{\sum_{d=1}^{D} S_{td}}{D}\#(1) \end{aligned}$$

Since PINS method cannot specify the number of clusters, we used the following criterion to calculate the number of significant log-rank p-values for the PINS method: “all possible numbers of clusters are significant if a cluster result (of a certain omics combination of certain cancer) is significant, and none are significant if a cluster result is not significant”. For the partially missing clustering results that exist in other methods, we calculate the parameters based on the assumption that the log-rank test of the missing results is significant.

Log-rank p-value distribution: Denote T as the number of cancer types, then the mean of the survival analysis p-values of a method across all clustering results for each cancer type, i.e., T means in total, forms the log-rank p-value distribution of this method.

**Supplementary Note 2: Evaluation Metrics**

Adjusted Rand Index (ARI): ARI measures the similarity between two clustering results. It corrects the Rand Index (RI) for the chance factor [1]. RI can be calculated as follows:

$$\begin{aligned} RI=\frac{a+b}{\left( \frac{n}{2} \right)}\#(2) \end{aligned}$$

where a is the number of pairs of elements that are in the same cluster in both clustering results, b is the number of pairs of elements that are in different clusters in both clustering results, and $(\frac{n}{2})$ is the total number of pairs of elements.

With RI calculated, ARI can be calculated as follows:

$$\begin{aligned} ARI=\frac{RI-E\left( RI \right)}{max\left( RI \right)-E\left( RI \right)}\#(3) \end{aligned}$$

where E(RI) is the expected RI value under a random model, and max(RI) is the maximum possible value of RI. ARI ranges from -0.5 to 1, with 1 indicating perfect agreement and 0 indicating no better than random.

Normalized Mutual Information (NMI): NMI measures the similarity between two clustering results by normalizing the Mutual Information (MI) by some generalized mean of the entropies of the two clustering results [2]. [MI is a measure of the statistical dependence between two random variables, and it is calculated as follows:](https://en.wikipedia.org/wiki/Mutual_information)

$$\begin{aligned} MI\left( U,V \right)=\sum_{u\in U} \sum_{v\in V} P\left( u,v \right)log \frac{P\left( u,v \right)}{P\left( u \right)P\left( v \right)}\#(4) \end{aligned}$$

where U and V are the true and predicted clustering results, respectively, P(u,v) is the joint probability distribution of U and V, and P(u) and P(v) are the marginal probability distributions of U and V, respectively. MI ranges from 0 to infinity, with 0 indicating that the two random variables are independent and higher values indicating higher dependence.

With MI calculated, [NMI can be calculated as follows:](https://en.wikipedia.org/wiki/Mutual_information)

$$\begin{aligned} NMI\left( U,V \right)=\frac{2\times MI\left( U,V \right)}{H\left( U \right)+H\left( V \right)}\#(5) \end{aligned}$$

where H(U) and H(V) are the entropies of U and V, respectively, and are calculated as:

$$\begin{aligned} H\left( U \right)=-\sum_{u\in U} P\left( u \right)log\left( P\left( u \right) \right)\#(6) \end{aligned}$$

$$\begin{aligned} H\left( V \right)=-\sum_{v\in V} P\left( v \right)\log\left( P\left( v \right) \right)\#(7) \end{aligned}$$

NMI ranges from 0 to 1, with 1 indicating perfect agreement and 0 indicating no mutual information.

Precision: Precision measures the proportion of true positives among the predicted positives. It reflects the accuracy of the prediction The calculation formula of Precision is TP/(TP+FP), where TP refers to true positive, FP refers to false positive.

Recall: the calculation formula is TP/(TP+FN), which represents the proportion of correct model recognition in positive class samples. FN refers to false negative.

F-score: F-score measures the harmonic mean of precision and recall. Recall measures the proportion of true positives among the actual positives. It reflects the completeness of the prediction.

$$\begin{aligned} F-score=\frac{2*Precision*Recall}{Precision+Recall}\#(8) \end{aligned}$$

Precision and F-score are metrics originally designed for classification, and to apply them to cluster evaluation, we need to establish the correspondence between the clusters obtained by clustering and the ground truth labels. We use the following method to establish the correspondence:

First, we construct a contingency matrix with the cluster labels as the row index and the ground truth labels as the column index, and each element in the matrix represents the number of samples in the corresponding cluster label that belong to a certain ground truth label, then we select the maximum element in the matrix, and assume that the labels of the row and column where the maximum element is located are matched, then we remove the row and column where the maximum element is located, and find the next maximum element. If there are multiple equal maximum elements, we choose the one with the smallest sum of the number of samples in the row and column where the maximum element is located, and repeat this process until the matrix is empty, then the correspondence is established.

After that, true positive (TP), false positive (FP), true negative (TN), false negative (FN) can be calculated as in the classification task, and then precision and F-score can be calculated.

We have verified that this calculation method is consistent with the precision calculation method in previous work [3], but we failed to reproduce the F-score reported in their work. Fortunately, they provided the contingency table data for all results, so we recalculated the F-score for each cluster result of each methods ourselves, using the following method:

$$\begin{aligned} F^{\left( i \right)}=\sum_{k=1}^{K} \frac{n_{k}}{N}F_{k}^{\left( i \right)},n_{1}+\ldots+n_{K}=N\#\left( 9 \right) \end{aligned}$$

Where $F^{\left( i \right)}$ represents the final F-score of i-th cluster result, K represents the number of clusters (5 for BRCA, 4 for COAD), $n_{k}$ represents the number of sample of k-th cluster, $F_{k}^{\left( i \right)}$ represents F-score of k-th cluster, which is calculated using Equation (8).

**Supplementary Figures：**


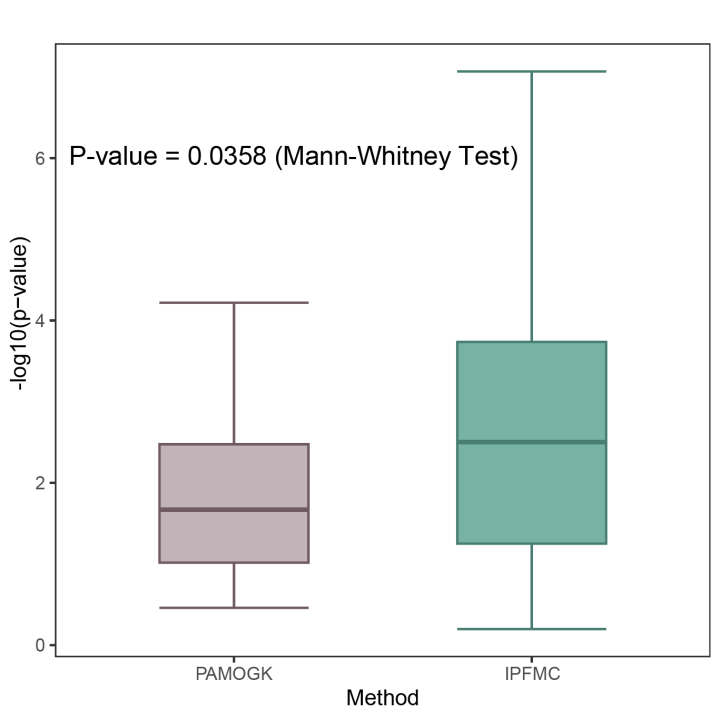


Supplementary Fig. 1 Performance of PAMOGK and IPFMC in survival analysis on KIRC.


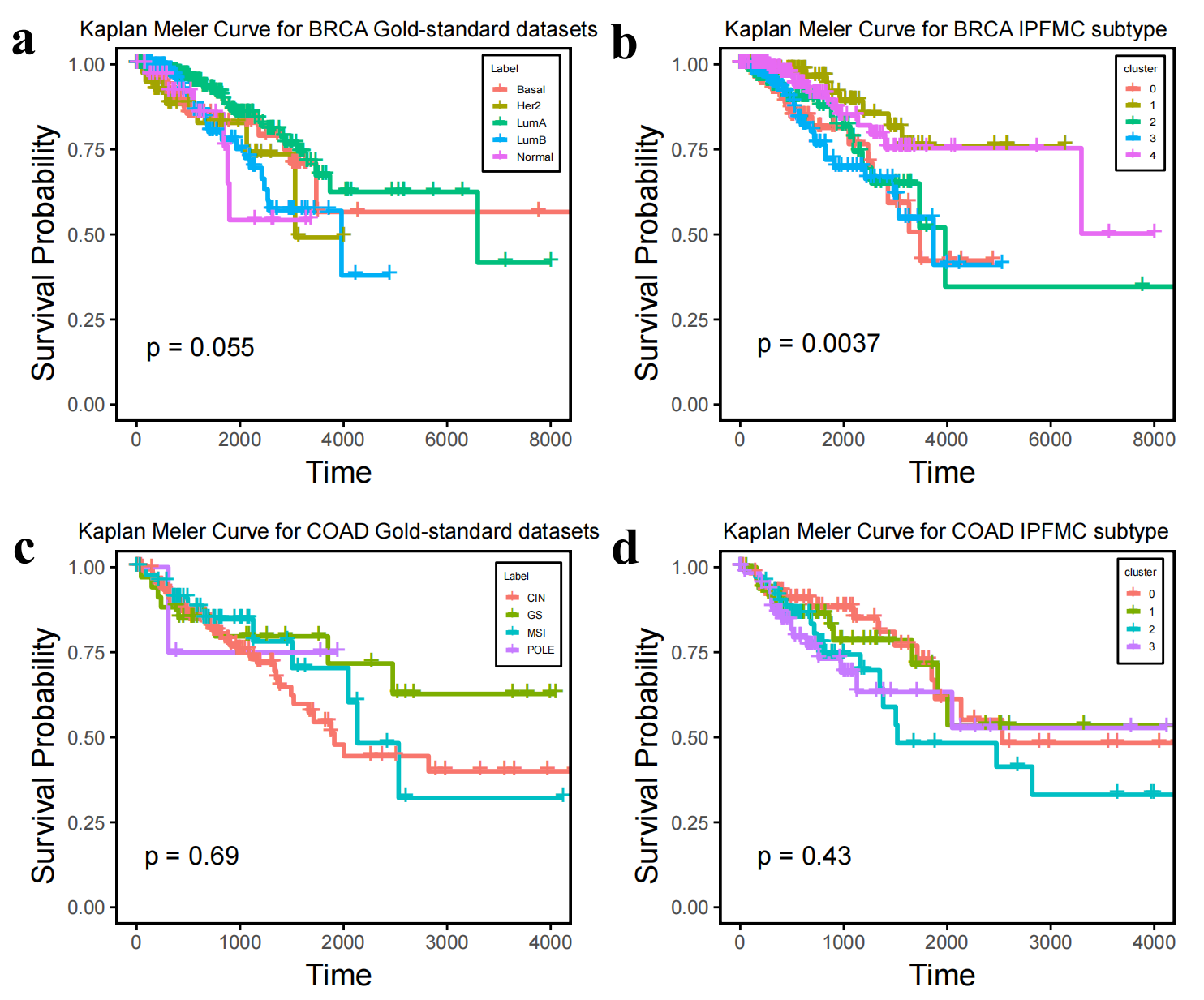


Supplementary Fig. 2 Comparison of Kaplan Meler Curve between true-labels of Gold-standard datasets and Subtypes obtained by IPFMC (strategy one).

**
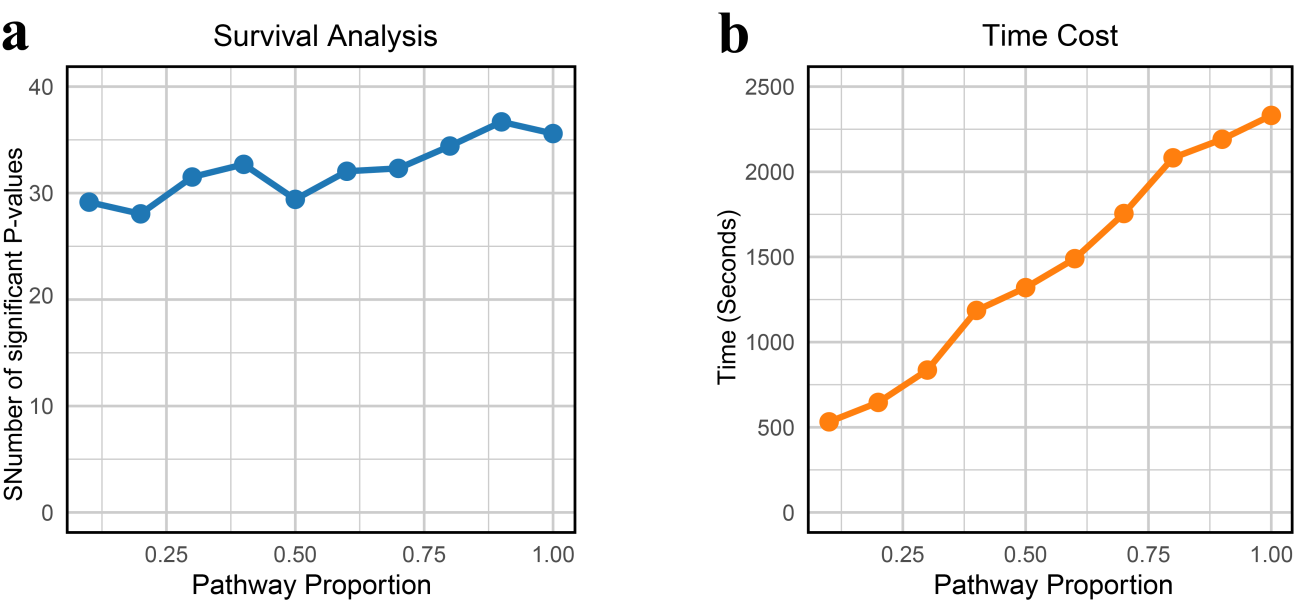
**

Supplementary Fig. 3 The relationship between survival analysis results (a), average runtime for Strategy 1 across 9 cancer types (b), and the proportion of pathway used in the model.

**Supplementary Tables：**

Supplementary Table 1. Survival analysis results (log-rank p-value) of IPFMC

| Cancer | Combination | Number of clusters | | | | | | |
| --- | --- | --- | --- | --- | --- | --- | --- | --- |
|  |  | 2 | 3 | 4 | 5 | 6 | 7 | 8 |
| BRCA | m_mi | 0.0096 | 0.0109 | 0.0332 | 0.0083 | 0.0835 | 0.0923 | 0.1164 |
|  | m_me | 0.0401 | 0.0749 | 0.1539 | 0.1412 | 0.1557 | 0.3329 | 0.3909 |
|  | m_cnv | 0.0098 | 0.0214 | 0.1260 | 0.1328 | 0.0956 | 0.0747 | 0.2546 |
|  | mi_me | 0.4284 | 0.0002 | 0.0014 | 0.0034 | 0.0518 | 0.0595 | 0.0511 |
|  | mi_cnv | 0.0009 | 0.3921 | 0.0061 | 0.0113 | 0.0064 | 0.0603 | 0.0418 |
|  | me_cnv | 0.0917 | 0.0324 | 0.0651 | 0.0470 | 0.0129 | 0.0134 | 0.0074 |
|  | m_mi_me | 0.0188 | 0.0989 | 0.0257 | 0.0074 | 0.0005 | 0.0362 | 0.3666 |
|  | m_mi_cnv | 0.0120 | 0.0152 | 0.0053 | 0.1142 | 0.0305 | 0.0868 | 0.1944 |
|  | m_me_cnv | 0.0054 | 0.0105 | 0.0060 | 0.0636 | 0.0223 | 0.1464 | 0.0644 |
|  | mi_me_cnv | 0.1458 | 0.3344 | 0.4030 | 0.0054 | 0.0109 | 0.0059 | 0.1289 |
|  | m_mi_me_cnv | 0.0012 | 0.1115 | 0.0192 | 0.0159 | 0.0140 | 0.0616 | 0.1296 |
| COAD | m_mi | 0.4104 | 0.2460 | 0.0263 | 0.0087 | 0.0316 | 0.0411 | 0.0400 |
|  | m_me | 0.6035 | 0.3839 | 0.2524 | 0.5379 | 0.4041 | 0.4496 | 0.2618 |
|  | m_cnv | 0.1348 | 0.3499 | 0.4658 | 0.0114 | 0.0395 | 0.0420 | 0.0543 |
|  | mi_me | 0.2164 | 0.2564 | 0.0334 | 0.0421 | 0.0491 | 0.0565 | 0.1222 |
|  | mi_cnv | 0.0950 | 0.0079 | 0.0501 | 0.0273 | 0.1523 | 0.1162 | 0.1255 |
|  | me_cnv | 0.0409 | 0.3120 | 0.2773 | 0.1666 | 0.1080 | 0.0349 | 0.0158 |
|  | m_mi_me | 0.4047 | 0.0055 | 0.0832 | 0.1700 | 0.0792 | 0.2254 | 0.5104 |
|  | m_mi_cnv | 0.5055 | 0.0117 | 0.0025 | 0.0029 | 0.0016 | 0.2536 | 0.0792 |
|  | m_me_cnv | 0.1269 | 0.0351 | 0.2933 | 0.6511 | 0.3720 | 0.0080 | 0.0749 |
|  | mi_me_cnv | 0.6391 | 0.2163 | 0.2718 | 0.1966 | 0.1227 | 0.0676 | 0.4025 |
|  | m_mi_me_cnv | 0.4806 | 0.0022 | 0.0273 | 0.0032 | 0.0046 | 0.0035 | 0.0888 |
| KIRC | m_mi | 3.22E-06 | 3.55E-06 | 5.24E-06 | 1.82E-05 | 1.72E-05 | 0.0001 | 0.0002 |
|  | m_me | 7.47E-05 | 0.0061 | 0.0125 | 0.0439 | 0.0063 | 4.82E-06 | 0.0003 |
|  | m_cnv | 0.0007 | 0.0215 | 0.0120 | 0.0002 | 7.22E-05 | 4.75E-05 | 0.0002 |
|  | mi_me | 0.1887 | 0.0097 | 0.0009 | 0.0001 | 0.0008 | 0.0008 | 0.0016 |
|  | mi_cnv | 0.2329 | 0.0001 | 6.91E-05 | 0.0002 | 0.0014 | 0.0001 | 0.0001 |
|  | me_cnv | 0.2624 | 0.3827 | 0.3621 | 0.2929 | 0.2902 | 0.0701 | 0.0007 |
|  | m_mi_me | 0.0018 | 0.0013 | 0.0007 | 2.76E-05 | 2.81E-05 | 0.0006 | 0.0006 |
|  | m_mi_cnv | 8.54E-08 | 0.0006 | 3.66E-05 | 0.0016 | 7.63E-06 | 3.14E-05 | 0.0003 |
|  | m_me_cnv | 0.1181 | 0.1852 | 0.0068 | 0.0046 | 0.0033 | 0.0004 | 2.13E-05 |
|  | mi_me_cnv | 0.4803 | 0.2174 | 0.0316 | 0.0183 | 0.0002 | 0.0008 | 0.0004 |
|  | m_mi_me_cnv | 0.0004 | 0.6346 | 0.0142 | 0.0021 | 0.0056 | 0.0037 | 0.0009 |
| LUAD | m_mi | 0.5254 | 0.0904 | 0.0408 | 0.0927 | 0.0452 | 0.0173 | 0.0028 |
|  | m_me | 0.0118 | 0.0010 | 0.0007 | 0.0078 | 0.0106 | 0.0214 | 0.0268 |
|  | m_cnv | 0.1125 | 0.3811 | 0.3122 | 0.3232 | 0.5871 | 0.3541 | 0.0630 |
|  | mi_me | 0.0118 | 0.0812 | 0.0424 | 0.0658 | 0.0777 | 0.0717 | 0.0164 |
|  | mi_cnv | 0.0089 | 0.0370 | 0.3726 | 0.0895 | 0.0886 | 0.0429 | 0.2067 |
|  | me_cnv | 0.3419 | 0.0010 | 0.0027 | 0.0260 | 0.5645 | 0.1987 | 0.2970 |
|  | m_mi_me | 0.7580 | 0.0758 | 0.0339 | 0.0099 | 0.0172 | 0.0207 | 0.0205 |
|  | m_mi_cnv | 0.1567 | 0.1972 | 0.3292 | 0.1603 | 0.1126 | 0.0788 | 0.2085 |
|  | m_me_cnv | 0.0325 | 0.0797 | 0.0081 | 0.2611 | 0.0278 | 0.2562 | 0.2736 |
|  | mi_me_cnv | 0.0507 | 0.0119 | 0.0039 | 0.0235 | 0.0073 | 0.0269 | 0.0521 |
|  | m_mi_me_cnv | 0.1082 | 0.1864 | 0.1371 | 0.1276 | 0.0031 | 0.0083 | 0.0115 |
| LUSC | m_mi | 0.8749 | 0.3632 | 0.1338 | 0.0176 | 0.1408 | 0.2922 | 0.6092 |
|  | m_me | 0.0177 | 0.0047 | 0.0314 | 0.0211 | 0.0289 | 0.0240 | 0.0454 |
|  | m_cnv | 0.5744 | 0.4358 | 0.5711 | 0.1060 | 0.2111 | 0.2268 | 0.4982 |
|  | mi_me | 0.0137 | 0.0172 | 0.0041 | 0.0091 | 0.0692 | 0.0260 | 0.0852 |
|  | mi_cnv | 0.7021 | 0.0987 | 0.1399 | 0.1277 | 0.0261 | 0.0700 | 0.1269 |
|  | me_cnv | 0.0033 | 0.0130 | 0.0623 | 0.1908 | 0.3317 | 0.4757 | 0.3861 |
|  | m_mi_me | 0.4394 | 0.0648 | 0.0127 | 0.0085 | 0.0192 | 0.0147 | 0.0057 |
|  | m_mi_cnv | 0.5917 | 0.3540 | 0.0183 | 0.0024 | 0.0037 | 0.0044 | 0.0029 |
|  | m_me_cnv | 0.3987 | 0.0301 | 0.0823 | 0.2370 | 0.3928 | 0.1347 | 0.2662 |
|  | mi_me_cnv | 0.8599 | 0.0032 | 0.0050 | 0.0090 | 0.0020 | 0.0008 | 0.0133 |
|  | m_mi_me_cnv | 0.8078 | 0.0338 | 0.0766 | 0.0004 | 0.0006 | 0.0106 | 0.0185 |
| ACC | m_mi | 0.0002 | 1.33E-05 | 6.29E-07 | 3.99E-07 | 5.51E-08 | 6.10E-06 | 0.0001 |
|  | m_me | 1.29E-06 | 0.0008 | 0.0001 | 0.0006 | 0.0012 | 1.40E-05 | 3.64E-11 |
|  | m_cnv | 0.0001 | 0.0006 | 0.0007 | 0.0021 | 0.0196 | 0.1221 | 0.0072 |
|  | mi_me | 0.0386 | 0.0004 | 0.0007 | 0.0008 | 7.14E-05 | 3.10E-05 | 0.0021 |
|  | mi_cnv | 0.0012 | 0.0007 | 0.0977 | 0.0844 | 0.1517 | 0.0243 | 0.0215 |
|  | me_cnv | 0.0002 | 1.33E-05 | 0.0006 | 0.0004 | 0.1063 | 0.1350 | 0.1110 |
|  | m_mi_me | 1.01E-07 | 5.48E-05 | 2.15E-08 | 4.11E-07 | 3.07E-07 | 6.68E-05 | 4.96E-06 |
|  | m_mi_cnv | 1.13E-05 | 0.0008 | 0.0028 | 8.07E-05 | 0.0003 | 0.0008 | 4.80E-05 |
|  | m_me_cnv | 4.78E-06 | 2.54E-06 | 1.54E-05 | 6.67E-07 | 0.0017 | 3.44E-05 | 0.0206 |
|  | mi_me_cnv | 1.92E-05 | 0.0012 | 0.0464 | 0.0005 | 0.1651 | 0.0012 | 0.0131 |
|  | m_mi_me_cnv | 5.69E-07 | 4.64E-07 | 2.48E-08 | 5.76E-07 | 5.10E-06 | 3.16E-05 | 4.34E-06 |
| KIRP | m_mi | 0.6995 | 0.7934 | 0.7175 | 0.3489 | 0.2702 | 0.4224 | 0.2076 |
|  | m_me | 0.4450 | 0.6656 | 0.0245 | 0.2262 | 0.2990 | 0.2066 | 0.0006 |
|  | m_cnv | 0.4450 | 0.6916 | 0.4237 | 0.6310 | 0.5272 | 0.0079 | 0.0104 |
|  | mi_me | 0.0002 | 0.0063 | 0.0002 | 0.0241 | 0.0003 | 0.0025 | 0.0201 |
|  | mi_cnv | 0.3850 | 0.7171 | 0.0282 | 0.0187 | 0.0129 | 0.0148 | 0.0385 |
|  | me_cnv | 1.31E-05 | 0.0005 | 9.16E-05 | 0.0003 | 0.0024 | 0.0005 | 1.24E-09 |
|  | m_mi_me | 0.4319 | 0.5546 | 0.8928 | 0.3857 | 0.2282 | 0.0339 | 0.0232 |
|  | m_mi_cnv | 0.2878 | 0.6800 | 0.4136 | 0.8917 | 0.8501 | 0.0973 | 0.1513 |
|  | m_me_cnv | 0.0002 | 0.0465 | 0.1614 | 0.1011 | 0.0006 | 0.0076 | 8.18E-05 |
|  | mi_me_cnv | 0.0004 | 0.0006 | 0.0004 | 0.0025 | 0.0023 | 0.0005 | 0.0183 |
|  | m_mi_me_cnv | 0.0108 | 0.2422 | 0.5457 | 0.8324 | 0.7335 | 0.0001 | 3.71E-05 |
| LIHC | m_mi | 0.5243 | 0.5574 | 0.5289 | 0.4606 | 0.2205 | 0.7710 | 0.6981 |
|  | m_me | 0.9423 | 0.9172 | 0.9880 | 0.6410 | 0.5095 | 0.1828 | 0.4688 |
|  | m_cnv | 0.4806 | 0.5579 | 0.5820 | 0.8992 | 0.4262 | 0.3118 | 0.4189 |
|  | mi_me | 0.8969 | 0.8492 | 0.9896 | 0.5334 | 0.4106 | 0.6545 | 0.7882 |
|  | mi_cnv | 0.8728 | 0.5259 | 0.2163 | 0.3598 | 0.1214 | 0.0241 | 0.0656 |
|  | me_cnv | 0.2802 | 0.1855 | 0.9285 | 0.7121 | 0.0965 | 0.3680 | 0.6744 |
|  | m_mi_me | 0.4992 | 0.4329 | 0.5119 | 0.9816 | 0.3588 | 0.8827 | 0.6506 |
|  | m_mi_cnv | 0.3477 | 0.4100 | 0.6383 | 0.6465 | 0.5819 | 0.0950 | 0.6112 |
|  | m_me_cnv | 0.2859 | 0.5784 | 0.4229 | 0.9123 | 0.5611 | 0.3701 | 0.6670 |
|  | mi_me_cnv | 0.3247 | 0.4317 | 0.4479 | 0.1740 | 0.7006 | 0.8417 | 0.4622 |
|  | m_mi_me_cnv | 0.5777 | 0.3748 | 0.1402 | 0.5436 | 0.4638 | 0.4999 | 0.9294 |
| THYM | m_mi | 0.0007 | 0.0014 | 0.0288 | 0.0021 | 0.0071 | 0.0167 | 0.0132 |
|  | m_me | 0.0014 | 0.0191 | 0.0389 | 0.0443 | 0.0444 | 0.0339 | 0.0604 |
|  | m_cnv | 0.0076 | 0.0133 | 0.0211 | 0.0462 | 0.0137 | 0.0107 | 0.0388 |
|  | mi_me | 0.0031 | 0.0117 | 0.0074 | 0.0015 | 0.0046 | 0.0095 | 0.0039 |
|  | mi_cnv | 2.84E-05 | 0.0002 | 0.0012 | 0.0013 | 0.0020 | 0.0068 | 0.0129 |
|  | me_cnv | 0.0004 | 0.0040 | 0.0130 | 0.0071 | 0.0014 | 0.0181 | 4.92E-05 |
|  | m_mi_me | 0.0041 | 0.0127 | 0.0035 | 0.0025 | 0.0005 | 0.0010 | 0.0121 |
|  | m_mi_cnv | 5.14E-05 | 0.0007 | 0.0004 | 0.0010 | 0.0024 | 0.0057 | 0.0028 |
|  | m_me_cnv | 0.0023 | 0.0190 | 0.0102 | 0.0139 | 0.0473 | 0.0150 | 0.0289 |
|  | mi_me_cnv | 0.0006 | 0.0005 | 0.0007 | 0.0016 | 0.0078 | 0.0096 | 0.0063 |
|  | m_mi_me_cnv | 0.0041 | 0.0061 | 0.0021 | 0.0054 | 0.0001 | 0.0007 | 0.0017 |

Notation: ‘m’ represents mRNA expression, ‘mi’ represents miRNA expression, ‘me’ represents methylation, and ‘cnv’ represents copy number variation. These notations remain consistent in the subsequent tables.

Supplementary Table 2. Wilcoxon test results between IPFMC and other methods on Survival analysis evaluation results

| Test method pair | P-values (Mann-Whitney U test) | |
| --- | --- | --- |
|  | Num of Sig | P-value distribution |
| IPF vs. iCB | 0.0028 | 0.0437 |
| IPF vs. LRA | 0.0024 | 0.0025 |
| IPF vs. SNF | 0.0471 | 0.2741 |
| IPF vs. PFA | 0.0022 | 0.0537 |
| IPF vs. NEMO | 0.0533 | 0.3169 |
| IPF vs. moC | 4.58E-05 | 0.0002 |
| IPF vs. CIM | 0.0219 | 0.0229 |
| IPF vs. MNMF | 0.0011 | 0.0061 |
| IPF vs. SGAN | 0.0455 | 0.1873 |
| IPF vs. PINS | 0.0676 | 0.0001 |

We use “iCB”, “LRA”, “moC”, “CIM”, “MNMF”, “SGAN” and “IPF” to represent iClusterBayes, LRAcluster, moCluster, CIMLR, MultiNMF, Subtype-GAN, IPFMC respectively.

Supplementary Table 3. Gold-standard datasets evaluation indicators of IPFMC

| Combination | BRCA |  |  |  | COAD |  |  |  |
| --- | --- | --- | --- | --- | --- | --- | --- | --- |
|  | ARI | NMI | Precision | F-score | ARI | NMI | Precision | F-score |
| m_mi | 0.2607 | 0.3331 | 0.5931 | 0.6183 | 0.1009 | 0.1009 | 0.4077 | 0.4802 |
| m_me | 0.3379 | 0.3238 | 0.4203 | 0.4484 | 0.3216 | 0.3216 | 0.5769 | 0.6366 |
| m_cnv | 0.2402 | 0.3289 | 0.5261 | 0.5649 | 0.3561 | 0.3561 | 0.5077 | 0.5847 |
| mi_me | 0.3536 | 0.3371 | 0.5291 | 0.5648 | 0.0939 | 0.0939 | 0.4462 | 0.5145 |
| mi_cnv | 0.2526 | 0.3385 | 0.4888 | 0.5151 | 0.6067 | 0.6067 | 0.5038 | 0.5906 |
| me_cnv | 0.1565 | 0.2058 | 0.5201 | 0.5544 | 0.3003 | 0.3003 | 0.5192 | 0.6028 |
| m_mi_me | 0.3556 | 0.3894 | 0.5306 | 0.5816 | 0.0853 | 0.0853 | 0.5346 | 0.5988 |
| m_mi_cnv | 0.2565 | 0.3435 | 0.5887 | 0.6265 | 0.4237 | 0.4237 | 0.5192 | 0.6011 |
| m_me_cnv | 0.3708 | 0.3564 | 0.6170 | 0.6448 | 0.4506 | 0.4506 | 0.5038 | 0.5822 |
| mi_me_cnv | 0.3908 | 0.3928 | 0.5976 | 0.6272 | 0.4949 | 0.4949 | 0.5538 | 0.6172 |
| m_mi_me_cnv | 0.3153 | 0.3967 | 0.6289 | 0.6564 | 0.4308 | 0.4308 | 0.4885 | 0.5634 |

Supplementary Table 4. F-score for gold-standard dataset evaluation of other methods

| Cancer | Combination | Method | | | | | | | | |
| --- | --- | --- | --- | --- | --- | --- | --- | --- | --- | --- |
|  |  | iCB | LRA | SNF | PFA | NEMO | moC | CIM | MNMF | SGAN |
| BRCA | m_mi | 0.4419 | 0.5529 | 0.5901 | 0.5568 | 0.6098 | 0.5145 | 0.5796 | 0.5205 | 0.5253 |
|  | m_me | 0.5157 | 0.5220 | 0.6380 | 0.4386 | 0.6460 | 0.3744 | 0.4994 | 0.5608 | 0.5757 |
|  | m_cnv | 0.5410 | 0.6135 | 0.6062 | 0.4601 | 0.5943 | 0.5394 | 0.6072 | 0.5237 | 0.5938 |
|  | mi_me | 0.4623 | 0.3300 | 0.6291 | 0.3834 | 0.6226 | 0.4432 | 0.5745 | 0.5423 | 0.5603 |
|  | mi_cnv | 0.4612 | 0.4633 | 0.6173 | 0.3787 | 0.5900 | 0.5159 | 0.5084 | 0.5620 | 0.4492 |
|  | me_cnv | 0.5138 | 0.3416 | 0.5931 | 0.3742 | 0.4947 | 0.3950 | 0.5857 | 0.3230 | 0.5029 |
|  | m_mi_me | 0.5076 | 0.5174 | 0.6093 | 0.4129 | 0.5908 | 0.4518 | 0.5558 | 0.6129 | 0.5477 |
|  | m_mi_cnv | 0.5672 | 0.6160 | 0.6212 | 0.4578 | 0.5872 | 0.5880 | 0.5767 | 0.5663 | 0.5696 |
|  | m_me_cnv | 0.4524 | 0.5699 | 0.6152 | 0.3719 | 0.6204 | 0.3627 | 0.5704 | 0.4979 | 0.5383 |
|  | mi_me_cnv | 0.4639 | 0.3365 | 0.6372 | 0.4344 | 0.6424 | 0.4875 | 0.5946 | 0.5461 | 0.6072 |
|  | m_mi_me_cnv | 0.4766 | 0.5744 | 0.6008 | 0.4857 | 0.6020 | 0.4158 | 0.6059 | 0.5662 | 0.4579 |
| COAD | m_mi | 0.5073 | 0.5289 | 0.5352 | 0.5879 | 0.5098 | 0.4238 | 0.4561 | 0.3863 | 0.4829 |
|  | m_me | 0.5859 | 0.5004 | 0.5140 | 0.5459 | 0.5550 | 0.4650 | 0.6187 | 0.4195 | 0.5464 |
|  | m_cnv | 0.4992 | 0.5928 | 0.6190 | 0.4297 | 0.5517 | 0.5031 | 0.6136 | 0.5253 | 0.4409 |
|  | mi_me | 0.4403 | 0.4735 | 0.5809 | 0.5627 | 0.5517 | 0.5345 | 0.4109 | 0.3830 | 0.4766 |
|  | mi_cnv | 0.4436 | 0.4946 | 0.6363 | 0.4438 | 0.6013 | 0.3599 | 0.4709 | 0.4187 | 0.4908 |
|  | me_cnv | 0.5046 | 0.5197 | 0.5872 | 0.5751 | 0.4758 | 0.5113 | 0.5696 | 0.4278 | 0.5707 |
|  | m_mi_me | 0.5793 | 0.5004 | 0.6184 | 0.4911 | 0.6626 | 0.6596 | 0.4504 | 0.4848 | 0.5007 |
|  | m_mi_cnv | 0.4740 | 0.5928 | 0.5723 | 0.3784 | 0.5791 | 0.4363 | 0.4776 | 0.4101 | 0.6396 |
|  | m_me_cnv | 0.6349 | 0.5274 | 0.5899 | 0.4855 | 0.6813 | 0.4999 | 0.5796 | 0.4032 | 0.6234 |
|  | mi_me_cnv | 0.4941 | 0.5232 | 0.5193 | 0.5417 | 0.6636 | 0.3897 | 0.4816 | 0.3946 | 0.4418 |
|  | m_mi_me_cnv | 0.6128 | 0.5294 | 0.5972 | 0.4854 | 0.5990 | 0.4115 | 0.4949 | 0.4724 | 0.4876 |

Notation: We use “iCB”, “LRA”, “moC”, “CIM”, “MNMF” and “SGAN” to represent iClusterBayes, LRAcluster, moCluster, CIMLR, MultiNMF and Subtype-GAN respectively.

Supplementary Table 5. K (number of clusters) used in gold-standard evaluation of IPFMC

| Combination | BRCA |  | COAD |  |
| --- | --- | --- | --- | --- |
|  | ARI, NMI | Precision, F-score | ARI, NMI | Precision, F-score |
| m_mi | 2 | 5 | 7 | 4 |
| m_me | 2 | 5 | 2 | 4 |
| m_cnv | 2 | 5 | 2 | 4 |
| mi_me | 2 | 5 | 4 | 4 |
| mi_cnv | 2 | 5 | 2 | 4 |
| me_cnv | 2 | 5 | 3 | 4 |
| m_mi_me | 2 | 5 | 2 | 4 |
| m_mi_cnv | 2 | 5 | 2 | 4 |
| m_me_cnv | 2 | 5 | 2 | 4 |
| mi_me_cnv | 2 | 5 | 2 | 4 |
| m_mi_me_cnv | 2 | 5 | 2 | 4 |

The value of ‘K’ for the ARI and NMI indicators is recommended by IPFMC, determined using the silhouette coefficient. For the precision and F-score indicators, ‘K’ is assigned a value of 5 for BRCA and 4 for COAD.

Supplementary Table 6. Pathway ranking of LUAD mRNA expression

| Rank | Pathway | Score (ARI) |
| --- | --- | --- |
| 1 | PID_MET_PATHWAY | 0.5960 |
| 2 | WP_ERBB_SIGNALING_PATHWAY | 0.5431 |
| 3 | WP_LEPTIN_SIGNALING_PATHWAY | 0.5235 |
| 4 | WP_GASTRIN_SIGNALING_PATHWAY | 0.5233 |
| 5 | PID_ERBB1_RECEPTOR_PROXIMAL_PATHWAY | 0.5201 |
| 6 | WP_REGULATION_OF_ACTIN_CYTOSKELETON | 0.5165 |
| 7 | KEGG_PROSTATE_CANCER | 0.5099 |
| 8 | KEGG_ERBB_SIGNALING_PATHWAY | 0.5099 |
| 9 | WP_MET_IN_TYPE_1_PAPILLARY_RENAL_CELL_CARCINOMA | 0.4858 |
| 10 | WP_NON_SMALL_CELL_LUNG_CANCER | 0.4767 |
| 11 | KEGG_NON_SMALL_CELL_LUNG_CANCER | 0.4651 |
| 12 | WP_HEPATOCYTE_GROWTH_FACTOR_RECEPTOR_SIGNALING | 0.4628 |
| 13 | PID_INSULIN_PATHWAY | 0.4616 |
| 14 | WP_ANGIOPOIETIN_LIKE_PROTEIN_8_REGULATORY_PATHWAY | 0.4611 |
| 15 | WP_GLIOBLASTOMA_SIGNALING_PATHWAYS | 0.4572 |
| 16 | WP_INSULIN_SIGNALING | 0.4545 |
| 17 | REACTOME_RAB_REGULATION_OF_TRAFFICKING | 0.4534 |
| 18 | PID_ERBB1_INTERNALIZATION_PATHWAY | 0.4519 |
| 19 | KEGG_CHRONIC_MYELOID_LEUKEMIA | 0.4482 |
| 20 | WP_MIRNA_REGULATION_OF_PROSTATE_CANCER_SIGNALING_PATHWAYS | 0.4467 |
| 21 | KEGG_ENDOMETRIAL_CANCER | 0.4437 |
| 22 | KEGG_COLORECTAL_CANCER | 0.4424 |
| 23 | BIOCARTA_MET_PATHWAY | 0.4381 |
| 24 | WP_ONCOSTATIN_M_SIGNALING_PATHWAY | 0.4368 |
| 25 | KEGG_ACUTE_MYELOID_LEUKEMIA | 0.4359 |
| 26 | BIOCARTA_MAPK_PATHWAY | 0.4336 |
| 27 | WP_THYROID_STIMULATING_HORMONE_TSH_SIGNALING_PATHWAY | 0.4303 |
| 28 | WP_PROLACTIN_SIGNALING_PATHWAY | 0.4299 |
| 29 | REACTOME_SIGNALING_BY_INSULIN_RECEPTOR | 0.4259 |
| 30 | REACTOME_TOLL_LIKE_RECEPTOR_9_TLR9_CASCADE | 0.4210 |
| 31 | REACTOME_RAB_GEFS_EXCHANGE_GTP_FOR_GDP_ON_RABS | 0.4208 |
| 32 | REACTOME_SIGNALING_BY_FGFR_IN_DISEASE | 0.4207 |
| 33 | KEGG_ENDOCYTOSIS | 0.4179 |
| 34 | KEGG_ADHERENS_JUNCTION | 0.4172 |
| 35 | WP_EGFR_TYROSINE_KINASE_INHIBITOR_RESISTANCE | 0.4172 |
| 36 | WP_EGF_EGFR_SIGNALING_PATHWAY | 0.4164 |
| 37 | PID_RET_PATHWAY | 0.4143 |
| 38 | REACTOME_MYD88_INDEPENDENT_TLR4_CASCADE | 0.4133 |
| 39 | PID_ERBB1_DOWNSTREAM_PATHWAY | 0.4099 |
| 40 | REACTOME_SIGNALING_BY_FGFR2 | 0.4091 |
| 41 | BIOCARTA_INSULIN_PATHWAY | 0.4086 |
| 42 | REACTOME_SIGNALING_BY_FGFR1_IN_DISEASE | 0.4072 |
| 43 | REACTOME_DEATH_RECEPTOR_SIGNALING | 0.4059 |
| 44 | WP_PDGF_PATHWAY | 0.4000 |
| 45 | WP_PHYSICO_CHEMICAL_FEATURES_AND_TOXICITY_ASSOCIATED_PATHWAYS | 0.3992 |
| 46 | WP_ENVELOPE_PROTEINS_AND_THEIR_POTENTIAL_ROLES_IN_EDMD_PHYSIOPATHOLOGY | 0.3977 |
| 47 | KEGG_INSULIN_SIGNALING_PATHWAY | 0.3962 |
| 48 | KEGG_MEDICUS_REFERENCE_EGF_EGFR_RAS_PI3K_SIGNALING_PATHWAY | 0.3959 |
| 49 | REACTOME_SIGNALING_BY_EGFR | 0.3939 |
| 50 | PID_VEGFR1_2_PATHWAY | 0.3920 |
| 51 | REACTOME_SIGNALING_BY_NTRKS | 0.3918 |
| 52 | REACTOME_SIGNALING_BY_VEGF | 0.3917 |
| 53 | KEGG_RENAL_CELL_CARCINOMA | 0.3890 |
| 54 | REACTOME_CONSTITUTIVE_SIGNALING_BY_LIGAND_RESPONSIVE_EGFR_CANCER_VARIANTS | 0.3890 |
| 55 | WP_MICRORNAS_IN_CARDIOMYOCYTE_HYPERTROPHY | 0.3887 |
| 56 | BIOCARTA_PYK2_PATHWAY | 0.3883 |
| 57 | REACTOME_RHOU_GTPASE_CYCLE | 0.3881 |
| 58 | PID_TRKR_PATHWAY | 0.3876 |
| 59 | WP_DNA_DAMAGE_RESPONSE_ONLY_ATM_DEPENDENT | 0.3874 |
| 60 | SIG_INSULIN_RECEPTOR_PATHWAY_IN_CARDIAC_MYOCYTES | 0.3870 |
| 61 | REACTOME_TOLL_LIKE_RECEPTOR_CASCADES | 0.3868 |
| 62 | WP_HEPATITIS_B_INFECTION | 0.3863 |
| 63 | KEGG_NEUROTROPHIN_SIGNALING_PATHWAY | 0.3858 |
| 64 | WP_TRANSLATION_INHIBITORS_IN_CHRONICALLY_ACTIVATED_PDGFRA_CELLS | 0.3858 |
| 65 | KEGG_MEDICUS_VARIANT_MUTATION_ACTIVATED_EGFR_TO_RAS_ERK_SIGNALING_PATHWAY | 0.3851 |
| 66 | REACTOME_RHOQ_GTPASE_CYCLE | 0.3844 |
| 67 | PID_IL6_7_PATHWAY | 0.3843 |
| 68 | WP_TNF_ALPHA_SIGNALING_PATHWAY | 0.3843 |
| 69 | PID_CDC42_PATHWAY | 0.3837 |
| 70 | REACTOME_RHOJ_GTPASE_CYCLE | 0.3824 |
| 71 | WP_TGF_BETA_SIGNALING_PATHWAY | 0.3806 |
| 72 | WP_MICROTUBULE_CYTOSKELETON_REGULATION | 0.3804 |
| 73 | KEGG_MEDICUS_VARIANT_AMPLIFIED_PDGFR_TO_RAS_ERK_SIGNALING_PATHWAY | 0.3794 |
| 74 | KEGG_MEDICUS_REFERENCE_EGF_EGFR_RAS_ERK_SIGNALING_PATHWAY | 0.3789 |
| 75 | SIG_CHEMOTAXIS | 0.3778 |
| 76 | PID_PDGFRB_PATHWAY | 0.3774 |
| 77 | BIOCARTA_EIF4_PATHWAY | 0.3764 |
| 78 | PID_TGFBR_PATHWAY | 0.3758 |
| 79 | WP_CARDIAC_HYPERTROPHIC_RESPONSE | 0.3756 |
| 80 | REACTOME_SIGNALING_BY_ERBB2_ECD_MUTANTS | 0.3753 |
| 81 | WP_MELANOMA | 0.3750 |
| 82 | WP_ENERGY_METABOLISM | 0.3748 |
| 83 | KEGG_GLIOMA | 0.3740 |
| 84 | KEGG_MEDICUS_VARIANT_MUTATION_ACTIVATED_MET_TO_RAS_ERK_SIGNALING_PATHWAY | 0.3739 |
| 85 | WP_ESTROGEN_SIGNALING_PATHWAY | 0.3731 |
| 86 | KEGG_MEDICUS_PATHOGEN_HBV_HBX_TO_RAS_ERK_SIGNALING_PATHWAY | 0.3731 |
| 87 | REACTOME_SIGNALING_BY_ERBB2 | 0.3727 |
| 88 | KEGG_PHOSPHATIDYLINOSITOL_SIGNALING_SYSTEM | 0.3721 |
| 89 | BIOCARTA_NGF_PATHWAY | 0.3720 |
| 90 | REACTOME_SYNTHESIS_OF_PIPS_AT_THE_PLASMA_MEMBRANE | 0.3715 |
| 91 | WP_BRAIN_DERIVED_NEUROTROPHIC_FACTOR_BDNF_SIGNALING_PATHWAY | 0.3714 |
| 92 | KEGG_MEDICUS_REFERENCE_FLT3LG_FLT3_RAS_ERK_SIGNALING_PATHWAY | 0.3705 |
| 93 | REACTOME_PI_METABOLISM | 0.3700 |
| 94 | REACTOME_SIGNALING_BY_FGFR1 | 0.3696 |
| 95 | WP_BREAST_CANCER_PATHWAY | 0.3689 |
| 96 | PID_ER_NONGENOMIC_PATHWAY | 0.3685 |
| 97 | WP_CAMKK2_PATHWAY | 0.3680 |
| 98 | KEGG_MEDICUS_VARIANT_IGF2_OVEREXPRESSION_TO_RAS_ERK_SIGNALING_PATHWAY | 0.3678 |
| 99 | REACTOME_CARGO_RECOGNITION_FOR_CLATHRIN_MEDIATED_ENDOCYTOSIS | 0.3674 |
| 100 | KEGG_MEDICUS_VARIANT_DUPLICATION_OR_MUTATION_ACTIVATED_FLT3_TO_RAS_ERK_SIGNALING_PATHWAY | 0.3666 |
| 101 | WP_PI3K_AKT_MTOR_SIGNALING_PATHWAY_AND_THERAPEUTIC_OPPORTUNITIES_IN_PROSTATE_CANCER | 0.3651 |
| 102 | REACTOME_MITOCHONDRIAL_BIOGENESIS | 0.3643 |
| 103 | REACTOME_SIGNALING_BY_FGFR | 0.3624 |
| 104 | SIG_PIP3_SIGNALING_IN_CARDIAC_MYOCTES | 0.3611 |
| 105 | WP_PANCREATIC_ADENOCARCINOMA_PATHWAY | 0.3604 |
| 106 | PID_LYSOPHOSPHOLIPID_PATHWAY | 0.3602 |
| 107 | KEGG_AXON_GUIDANCE | 0.3598 |
| 108 | PID_SMAD2_3NUCLEAR_PATHWAY | 0.3590 |
| 109 | REACTOME_TNF_SIGNALING | 0.3586 |
| 110 | KEGG_MEDICUS_REFERENCE_TGFA_EGFR_RAS_ERK_SIGNALING_PATHWAY | 0.3561 |
| 111 | KEGG_MEDICUS_REFERENCE_P4_PR_RAS_ERK_SIGNALING_PATHWAY | 0.3560 |
| 112 | REACTOME_P75_NTR_RECEPTOR_MEDIATED_SIGNALLING | 0.3554 |
| 113 | REACTOME_CELL_DEATH_SIGNALLING_VIA_NRAGE_NRIF_AND_NADE | 0.3549 |
| 114 | WP_PHOSPHOINOSITIDES_METABOLISM | 0.3545 |
| 115 | BIOCARTA_PDGF_PATHWAY | 0.3542 |
| 116 | KEGG_MEDICUS_REFERENCE_IGF_IGF1R_RAS_ERK_SIGNALING_PATHWAY | 0.3540 |
| 117 | KEGG_MEDICUS_VARIANT_AMPLIFIED_FGFR_TO_RAS_ERK_SIGNALING_PATHWAY | 0.3539 |
| 118 | PID_PI3K_PLC_TRK_PATHWAY | 0.3539 |
| 119 | KEGG_MEDICUS_VARIANT_MUTATION_INACTIVATED_FGF17_TO_RAS_ERK_SIGNALING_PATHWAY | 0.3534 |
| 120 | KEGG_MEDICUS_VARIANT_DUPLICATION_OR_MUTATION_ACTIVATED_FLT3_TO_RAS_PI3K_SIGNALING_PATHWAY | 0.3523 |
| 121 | WP_AGE_RAGE_PATHWAY | 0.3513 |
| 122 | KEGG_MEDICUS_VARIANT_BCR_ABL_FUSION_KINASE_TO_RAS_ERK_SIGNALING_PATHWAY | 0.3498 |
| 123 | KEGG_MEDICUS_PATHOGEN_HCMV_GB_TO_PDGFR_RAS_ERK_SIGNALING_PATHWAY | 0.3495 |
| 124 | SA_TRKA_RECEPTOR | 0.3479 |
| 125 | REACTOME_SIGNALING_BY_TGF_BETA_RECEPTOR_COMPLEX | 0.3477 |
| 126 | KEGG_MEDICUS_REFERENCE_CA2_PYK2_RAS_ERK_SIGNALING_PATHWAY | 0.3464 |
| 127 | KEGG_APOPTOSIS | 0.3458 |
| 128 | BIOCARTA_NFAT_PATHWAY | 0.3447 |
| 129 | KEGG_PANCREATIC_CANCER | 0.3440 |
| 130 | WP_NOTCH_SIGNALING_PATHWAY | 0.3433 |
| 131 | KEGG_MEDICUS_REFERENCE_HGF_MET_RAS_ERK_SIGNALING_PATHWAY | 0.3407 |
| 132 | REACTOME_SIGNALLING_TO_ERKS | 0.3369 |

Supplementary Table 7. Pathway ranking of LUAD miRNA expression

| Rank | Pathway | Score (ARI) |
| --- | --- | --- |
| 1 | KEGG_MELANOMA | 0.7932 |
| 2 | WP_HEPATITIS_B_INFECTION | 0.7482 |
| 3 | REACTOME_EXTRA_NUCLEAR_ESTROGEN_SIGNALING | 0.7480 |
| 4 | WP_GLIOBLASTOMA_SIGNALING_PATHWAYS | 0.7385 |
| 5 | WP_NON_SMALL_CELL_LUNG_CANCER | 0.7348 |
| 6 | WP_SMALL_CELL_LUNG_CANCER | 0.7347 |
| 7 | KEGG_NON_SMALL_CELL_LUNG_CANCER | 0.7256 |
| 8 | WP_UROTENSIN_II_MEDIATED_SIGNALING_PATHWAY | 0.7246 |
| 9 | WP_MIRNA_REGULATION_OF_PROSTATE_CANCER_SIGNALING_PATHWAYS | 0.7227 |
| 10 | BIOCARTA_ERK_PATHWAY | 0.7142 |
| 11 | KEGG_PANCREATIC_CANCER | 0.7096 |
| 12 | WP_TGF_BETA_RECEPTOR_SIGNALING_IN_SKELETAL_DYSPLASIAS | 0.7059 |
| 13 | PID_IFNG_PATHWAY | 0.6989 |
| 14 | WP_CHROMOSOMAL_AND_MICROSATELLITE_INSTABILITY_IN_COLORECTAL_CANCER | 0.6986 |
| 15 | WP_ARYL_HYDROCARBON_RECEPTOR_PATHWAY_WP2586 | 0.6981 |
| 16 | WP_BRAIN_DERIVED_NEUROTROPHIC_FACTOR_BDNF_SIGNALING_PATHWAY | 0.6961 |
| 17 | WP_GROWTH_FACTORS_AND_HORMONES_IN_BETA_CELL_PROLIFERATION | 0.6953 |
| 18 | KEGG_FOCAL_ADHESION | 0.6933 |
| 19 | WP_FOCAL_ADHESION | 0.6922 |
| 20 | PID_PDGFRB_PATHWAY | 0.6853 |
| 21 | PID_AP1_PATHWAY | 0.6837 |
| 22 | WP_EGFR_TYROSINE_KINASE_INHIBITOR_RESISTANCE | 0.6815 |
| 23 | WP_PHOTODYNAMIC_THERAPY_INDUCED_AP_1_SURVIVAL_SIGNALING | 0.6795 |
| 24 | WP_TGF_BETA_RECEPTOR_SIGNALING | 0.6794 |
| 25 | REACTOME_INTRINSIC_PATHWAY_FOR_APOPTOSIS | 0.6790 |
| 26 | KEGG_GLIOMA | 0.6774 |
| 27 | PID_AVB3_OPN_PATHWAY | 0.6706 |
| 28 | REACTOME_SIGNALING_BY_TGF_BETA_RECEPTOR_COMPLEX | 0.6698 |
| 29 | BIOCARTA_P53_PATHWAY | 0.6648 |
| 30 | KEGG_CHRONIC_MYELOID_LEUKEMIA | 0.6593 |
| 31 | KEGG_COLORECTAL_CANCER | 0.6580 |
| 32 | KEGG_RENAL_CELL_CARCINOMA | 0.6528 |
| 33 | WP_B_CELL_RECEPTOR_SIGNALING_PATHWAY | 0.6514 |
| 34 | BIOCARTA_HCMV_PATHWAY | 0.6496 |
| 35 | WP_TROP2_REGULATORY_SIGNALING | 0.6483 |
| 36 | KEGG_PROSTATE_CANCER | 0.6472 |
| 37 | WP_LEPTIN_SIGNALING_PATHWAY | 0.6472 |
| 38 | WP_EPITHELIAL_TO_MESENCHYMAL_TRANSITION_IN_COLORECTAL_CANCER | 0.6471 |
| 39 | WP_HEAD_AND_NECK_SQUAMOUS_CELL_CARCINOMA | 0.6450 |
| 40 | WP_HEPATITIS_C_AND_HEPATOCELLULAR_CARCINOMA | 0.6413 |
| 41 | PID_BCR_5PATHWAY | 0.6377 |
| 42 | WP_HEPATOCYTE_GROWTH_FACTOR_RECEPTOR_SIGNALING | 0.6375 |
| 43 | PID_AVB3_INTEGRIN_PATHWAY | 0.6371 |
| 44 | WP_TNF_ALPHA_SIGNALING_PATHWAY | 0.6328 |
| 45 | BIOCARTA_LONGEVITY_PATHWAY | 0.6315 |
| 46 | REACTOME_FLT3_SIGNALING | 0.6314 |
| 47 | WP_ACUTE_VIRAL_MYOCARDITIS | 0.6309 |
| 48 | PID_MYC_REPRESS_PATHWAY | 0.6229 |
| 49 | PID_KIT_PATHWAY | 0.6227 |
| 50 | WP_BLADDER_CANCER | 0.6199 |
| 51 | SIG_IL4RECEPTOR_IN_B_LYPHOCYTES | 0.6178 |
| 52 | WP_PDGF_PATHWAY | 0.6173 |
| 53 | PID_CMYB_PATHWAY | 0.6163 |
| 54 | PID_ATF2_PATHWAY | 0.6158 |
| 55 | WP_ANDROGEN_RECEPTOR_NETWORK_IN_PROSTATE_CANCER | 0.6145 |
| 56 | WP_NEUROINFLAMMATION_AND_GLUTAMATERGIC_SIGNALING | 0.6136 |
| 57 | KEGG_ENDOMETRIAL_CANCER | 0.6130 |
| 58 | WP_ERK_PATHWAY_IN_HUNTINGTON_39_S_DISEASE | 0.6129 |
| 59 | REACTOME_SIGNALING_BY_ERBB2 | 0.6091 |
| 60 | PID_PI3K_PLC_TRK_PATHWAY | 0.6087 |
| 61 | PID_ERBB2_ERBB3_PATHWAY | 0.6068 |
| 62 | KEGG_ERBB_SIGNALING_PATHWAY | 0.6062 |
| 63 | REACTOME_REGULATION_OF_LOCALIZATION_OF_FOXO_TRANSCRIPTION_FACTORS | 0.6049 |
| 64 | WP_PANCREATIC_ADENOCARCINOMA_PATHWAY | 0.6017 |
| 65 | WP_INTERLEUKIN_11_SIGNALING_PATHWAY | 0.6015 |
| 66 | KEGG_SMALL_CELL_LUNG_CANCER | 0.6010 |
| 67 | BIOCARTA_TFF_PATHWAY | 0.6009 |
| 68 | BIOCARTA_NTHI_PATHWAY | 0.6002 |
| 69 | WP_AGE_RAGE_PATHWAY | 0.6002 |
| 70 | WP_PRION_DISEASE_PATHWAY | 0.5985 |
| 71 | WP_MICRORNAS_IN_CARDIOMYOCYTE_HYPERTROPHY | 0.5981 |
| 72 | WP_CKAP4_SIGNALING_PATHWAY_MAP | 0.5970 |
| 73 | WP_GASTRIN_SIGNALING_PATHWAY | 0.5945 |
| 74 | WP_ENDOMETRIAL_CANCER | 0.5916 |
| 75 | WP_EMBRYONIC_STEM_CELL_PLURIPOTENCY_PATHWAYS | 0.5912 |
| 76 | WP_ERBB_SIGNALING_PATHWAY | 0.5908 |
| 77 | WP_EXTRACELLULAR_VESICLE_MEDIATED_SIGNALING_IN_RECIPIENT_CELLS | 0.5904 |
| 78 | WP_MELANOMA | 0.5896 |
| 79 | WP_MEASLES_VIRUS_INFECTION | 0.5887 |
| 80 | WP_RAC1_PAK1_P38_MMP2_PATHWAY | 0.5883 |
| 81 | BIOCARTA_BAD_PATHWAY | 0.5872 |
| 82 | PID_P53_DOWNSTREAM_PATHWAY | 0.5856 |
| 83 | WP_SYNAPTIC_SIGNALING_PATHWAYS_ASSOCIATED_WITH_AUTISM_SPECTRUM_DISORDER | 0.5846 |
| 84 | PID_IL2_PI3K_PATHWAY | 0.5837 |
| 85 | PID_CERAMIDE_PATHWAY | 0.5836 |
| 86 | WP_PROLACTIN_SIGNALING_PATHWAY | 0.5825 |
| 87 | WP_ADIPOGENESIS | 0.5822 |
| 88 | WP_H19_ACTION_RB_E2F1_SIGNALING_AND_CDK_BETA_CATENIN_ACTIVITY | 0.5819 |
| 89 | BIOCARTA_NFAT_PATHWAY | 0.5804 |
| 90 | BIOCARTA_TEL_PATHWAY | 0.5790 |
| 91 | REACTOME_INTERLEUKIN_4_AND_INTERLEUKIN_13_SIGNALING | 0.5739 |
| 92 | PID_MET_PATHWAY | 0.5726 |
| 93 | BIOCARTA_PDGF_PATHWAY | 0.5723 |
| 94 | WP_INTEGRATED_CANCER_PATHWAY | 0.5706 |
| 95 | PID_TRKR_PATHWAY | 0.5702 |
| 96 | REACTOME_ESTROGEN_DEPENDENT_NUCLEAR_EVENTS_DOWNSTREAM_OF_ESR_MEMBRANE_SIGNALING | 0.5698 |
| 97 | WP_RELATIONSHIP_BETWEEN_INFLAMMATION_COX_2_AND_EGFR | 0.5696 |
| 98 | SIG_PIP3_SIGNALING_IN_CARDIAC_MYOCTES | 0.5673 |
| 99 | WP_IL_5_SIGNALING_PATHWAY | 0.5672 |
| 100 | KEGG_MEDICUS_REFERENCE_EREG_EGFR_PI3K_SIGNALING_PATHWAY | 0.5667 |
| 101 | WP_ONCOSTATIN_M_SIGNALING_PATHWAY | 0.5639 |
| 102 | BIOCARTA_HER2_PATHWAY | 0.5637 |
| 103 | WP_PHOTODYNAMIC_THERAPY_INDUCED_HIF_1_SURVIVAL_SIGNALING | 0.5617 |
| 104 | PID_FAK_PATHWAY | 0.5612 |
| 105 | BIOCARTA_MET_PATHWAY | 0.5610 |
| 106 | WP_WNT_SIGNALING_PATHWAY | 0.5602 |
| 107 | WP_A_NETWORK_MAP_OF_MACROPHAGE_STIMULATING_PROTEIN_MSP_SIGNALING | 0.5576 |
| 108 | WP_EGF_EGFR_SIGNALING_PATHWAY | 0.5557 |
| 109 | WP_INTERFERON_TYPE_I_SIGNALING_PATHWAYS | 0.5548 |
| 110 | REACTOME_CONSTITUTIVE_SIGNALING_BY_EGFRVIII | 0.5548 |
| 111 | REACTOME_KEAP1_NFE2L2_PATHWAY | 0.5537 |
| 112 | REACTOME_CONSTITUTIVE_SIGNALING_BY_AKT1_E17K_IN_CANCER | 0.5536 |
| 113 | WP_THYROID_STIMULATING_HORMONE_TSH_SIGNALING_PATHWAY | 0.5515 |
| 114 | PID_EPHB_FWD_PATHWAY | 0.5504 |
| 115 | REACTOME_ONCOGENIC_MAPK_SIGNALING | 0.5496 |
| 116 | WP_BREAST_CANCER_PATHWAY | 0.5495 |
| 117 | REACTOME_TP53_REGULATES_METABOLIC_GENES | 0.5482 |
| 118 | KEGG_MEDICUS_ENV_FACTOR_NNK_NNN_TO_RAS_ERK_SIGNALING_PATHWAY | 0.5481 |
| 119 | REACTOME_SIGNALING_BY_NTRKS | 0.5457 |
| 120 | KEGG_ACUTE_MYELOID_LEUKEMIA | 0.5450 |
| 121 | WP_MET_IN_TYPE_1_PAPILLARY_RENAL_CELL_CARCINOMA | 0.5440 |
| 122 | REACTOME_SIGNALING_BY_VEGF | 0.5400 |
| 123 | REACTOME_CELLULAR_SENESCENCE | 0.5398 |
| 124 | WP_NEOVASCULARISATION_PROCESSES | 0.5381 |
| 125 | BIOCARTA_IL6_PATHWAY | 0.5311 |
| 126 | PID_RB_1PATHWAY | 0.5304 |
| 127 | WP_CORTICOTROPIN_RELEASING_HORMONE_SIGNALING_PATHWAY | 0.5302 |
| 128 | PID_IL2_1PATHWAY | 0.5272 |
| 129 | REACTOME_SIGNALING_BY_BRAF_AND_RAF1_FUSIONS | 0.5119 |

Supplementary Table 8. Pathway ranking of LUAD methylation

| Rank | Pathway | Score (ARI) |
| --- | --- | --- |
| 1 | REACTOME_HOST_INTERACTIONS_OF_HIV_FACTORS | 0.8429 |
| 2 | REACTOME_SNRNP_ASSEMBLY | 0.8404 |
| 3 | REACTOME_G2_M_CHECKPOINTS | 0.8390 |
| 4 | REACTOME_TRANSPORT_OF_MATURE_TRANSCRIPT_TO_CYTOPLASM | 0.8328 |
| 5 | REACTOME_TRANSPORT_OF_MATURE_MRNAS_DERIVED_FROM_INTRONLESS_TRANSCRIPTS | 0.8279 |
| 6 | REACTOME_SEPARATION_OF_SISTER_CHROMATIDS | 0.8264 |
| 7 | REACTOME_METABOLISM_OF_POLYAMINES | 0.8247 |
| 8 | REACTOME_REGULATION_OF_GLUCOKINASE_BY_GLUCOKINASE_REGULATORY_PROTEIN | 0.8111 |
| 9 | REACTOME_NUCLEAR_PORE_COMPLEX_NPC_DISASSEMBLY | 0.8053 |
| 10 | REACTOME_INTERACTIONS_OF_REV_WITH_HOST_CELLULAR_PROTEINS | 0.8000 |
| 11 | REACTOME_ISG15_ANTIVIRAL_MECHANISM | 0.7987 |
| 12 | REACTOME_MITOTIC_SPINDLE_CHECKPOINT | 0.7978 |
| 13 | REACTOME_MAPK6_MAPK4_SIGNALING | 0.7921 |
| 14 | REACTOME_RESOLUTION_OF_SISTER_CHROMATID_COHESION | 0.7898 |
| 15 | REACTOME_TRANSCRIPTIONAL_REGULATION_BY_SMALL_RNAS | 0.7866 |
| 16 | REACTOME_CYCLIN_A_CDK2_ASSOCIATED_EVENTS_AT_S_PHASE_ENTRY | 0.7837 |
| 17 | REACTOME_TRNA_PROCESSING_IN_THE_NUCLEUS | 0.7806 |
| 18 | REACTOME_REGULATION_OF_RAS_BY_GAPS | 0.7732 |
| 19 | REACTOME_SUMOYLATION_OF_DNA_DAMAGE_RESPONSE_AND_REPAIR_PROTEINS | 0.7724 |
| 20 | REACTOME_SUMOYLATION_OF_UBIQUITINYLATION_PROTEINS | 0.7721 |
| 21 | REACTOME_SUMOYLATION_OF_SUMOYLATION_PROTEINS | 0.7706 |
| 22 | REACTOME_NUCLEAR_IMPORT_OF_REV_PROTEIN | 0.7705 |
| 23 | REACTOME_ANTIGEN_PROCESSING_CROSS_PRESENTATION | 0.7684 |
| 24 | REACTOME_REGULATION_OF_MRNA_STABILITY_BY_PROTEINS_THAT_BIND_AU_RICH_ELEMENTS | 0.7675 |
| 25 | REACTOME_TRANSPORT_OF_THE_SLBP_DEPENDANT_MATURE_MRNA | 0.7657 |
| 26 | REACTOME_ORC1_REMOVAL_FROM_CHROMATIN | 0.7654 |
| 27 | REACTOME_PTEN_REGULATION | 0.7648 |
| 28 | REACTOME_INTERACTIONS_OF_VPR_WITH_HOST_CELLULAR_PROTEINS | 0.7634 |
| 29 | REACTOME_UCH_PROTEINASES | 0.7627 |
| 30 | REACTOME_APC_C_MEDIATED_DEGRADATION_OF_CELL_CYCLE_PROTEINS | 0.7603 |
| 31 | REACTOME_SUMOYLATION_OF_DNA_REPLICATION_PROTEINS | 0.7596 |
| 32 | REACTOME_ACTIVATION_OF_THE_MRNA_UPON_BINDING_OF_THE_CAP_BINDING_COMPLEX_AND_EIFS_AND_SUBSEQUENT_BINDING_TO_43S | 0.7566 |
| 33 | REACTOME_GLYCOLYSIS | 0.7565 |
| 34 | REACTOME_ACTIVATION_OF_ATR_IN_RESPONSE_TO_REPLICATION_STRESS | 0.7552 |
| 35 | REACTOME_APC_C_CDH1_MEDIATED_DEGRADATION_OF_CDC20_AND_OTHER_APC_C_CDH1_TARGETED_PROTEINS_IN_LATE_MITOSIS_EARLY_G1 | 0.7539 |
| 36 | REACTOME_VIRAL_MESSENGER_RNA_SYNTHESIS | 0.7537 |
| 37 | REACTOME_SCF_BETA_TRCP_MEDIATED_DEGRADATION_OF_EMI1 | 0.7513 |
| 38 | REACTOME_RUNX1_REGULATES_TRANSCRIPTION_OF_GENES_INVOLVED_IN_DIFFERENTIATION_OF_HSCS | 0.7464 |
| 39 | REACTOME_G1_S_DNA_DAMAGE_CHECKPOINTS | 0.7463 |
| 40 | REACTOME_ANTIVIRAL_MECHANISM_BY_IFN_STIMULATED_GENES | 0.7457 |
| 41 | REACTOME_REGULATION_OF_PTEN_STABILITY_AND_ACTIVITY | 0.7447 |
| 42 | REACTOME_HCMV_EARLY_EVENTS | 0.7388 |
| 43 | REACTOME_CELLULAR_RESPONSE_TO_HYPOXIA | 0.7364 |
| 44 | REACTOME_EXPORT_OF_VIRAL_RIBONUCLEOPROTEINS_FROM_NUCLEUS | 0.7321 |
| 45 | REACTOME_SUMOYLATION_OF_RNA_BINDING_PROTEINS | 0.7311 |
| 46 | KEGG_MEDICUS_REFERENCE_NUCLEAR_EXPORT_OF_MRNA | 0.7255 |
| 47 | REACTOME_NEGATIVE_REGULATION_OF_NOTCH4_SIGNALING | 0.7255 |
| 48 | REACTOME_SWITCHING_OF_ORIGINS_TO_A_POST_REPLICATIVE_STATE | 0.7245 |
| 49 | REACTOME_RHO_GTPASES_ACTIVATE_FORMINS | 0.7215 |
| 50 | REACTOME_DEGRADATION_OF_GLI1_BY_THE_PROTEASOME | 0.7197 |
| 51 | REACTOME_NS1_MEDIATED_EFFECTS_ON_HOST_PATHWAYS | 0.7197 |
| 52 | WP_PROTEASOME_DEGRADATION | 0.7190 |
| 53 | REACTOME_MITOTIC_G1_PHASE_AND_G1_S_TRANSITION | 0.7186 |
| 54 | REACTOME_REGULATION_OF_RUNX2_EXPRESSION_AND_ACTIVITY | 0.7170 |
| 55 | REACTOME_HCMV_INFECTION | 0.7167 |
| 56 | REACTOME_TRNA_PROCESSING | 0.7162 |
| 57 | KEGG_SPLICEOSOME | 0.7155 |
| 58 | REACTOME_RNA_POLYMERASE_II_TRANSCRIPTION_TERMINATION | 0.7144 |
| 59 | REACTOME_THE_ROLE_OF_GTSE1_IN_G2_M_PROGRESSION_AFTER_G2_CHECKPOINT | 0.7144 |
| 60 | REACTOME_THE_CITRIC_ACID_TCA_CYCLE_AND_RESPIRATORY_ELECTRON_TRANSPORT | 0.7131 |
| 61 | REACTOME_HEDGEHOG_ON_STATE | 0.7129 |
| 62 | REACTOME_HEDGEHOG_LIGAND_BIOGENESIS | 0.7104 |
| 63 | REACTOME_REGULATION_OF_RUNX3_EXPRESSION_AND_ACTIVITY | 0.7080 |
| 64 | REACTOME_NUCLEAR_ENVELOPE_BREAKDOWN | 0.7076 |
| 65 | REACTOME_STABILIZATION_OF_P53 | 0.7073 |
| 66 | REACTOME_G1_S_SPECIFIC_TRANSCRIPTION | 0.7067 |
| 67 | REACTOME_DEGRADATION_OF_DVL | 0.7063 |
| 68 | REACTOME_S_PHASE | 0.7063 |
| 69 | REACTOME_CROSS_PRESENTATION_OF_SOLUBLE_EXOGENOUS_ANTIGENS_ENDOSOMES | 0.7044 |
| 70 | KEGG_PROTEASOME | 0.7043 |
| 71 | REACTOME_SUMOYLATION_OF_CHROMATIN_ORGANIZATION_PROTEINS | 0.7038 |
| 72 | REACTOME_NUCLEAR_EVENTS_MEDIATED_BY_NFE2L2 | 0.7036 |
| 73 | REACTOME_INTRA_GOLGI_TRAFFIC | 0.7035 |
| 74 | REACTOME_SCF_SKP2_MEDIATED_DEGRADATION_OF_P27_P21 | 0.7020 |
| 75 | PID_FOXM1_PATHWAY | 0.7011 |
| 76 | REACTOME_INFLUENZA_INFECTION | 0.7003 |
| 77 | REACTOME_AUF1_HNRNP_D0_BINDS_AND_DESTABILIZES_MRNA | 0.6990 |
| 78 | KEGG_RNA_POLYMERASE | 0.6983 |
| 79 | REACTOME_PROCESSING_OF_INTRONLESS_PRE_MRNAS | 0.6972 |
| 80 | REACTOME_COPI_DEPENDENT_GOLGI_TO_ER_RETROGRADE_TRAFFIC | 0.6928 |
| 81 | REACTOME_DOWNSTREAM_SIGNALING_EVENTS_OF_B_CELL_RECEPTOR_BCR | 0.6917 |
| 82 | REACTOME_SIGNALING_BY_NOTCH4 | 0.6913 |
| 83 | REACTOME_RESOLUTION_OF_ABASIC_SITES_AP_SITES | 0.6904 |
| 84 | REACTOME_FCERI_MEDIATED_NF_KB_ACTIVATION | 0.6904 |
| 85 | REACTOME_DEFECTIVE_CFTR_CAUSES_CYSTIC_FIBROSIS | 0.6895 |
| 86 | REACTOME_TRANSCRIPTIONAL_REGULATION_BY_RUNX2 | 0.6878 |
| 87 | PID_ATM_PATHWAY | 0.6864 |
| 88 | KEGG_MEDICUS_REFERENCE_ACTIVATION_OF_PRC2.2_BY_UBIQUITINATION_OF_H2AK119_IN_GERMLINE_GENES | 0.6851 |
| 89 | REACTOME_INTERLEUKIN_1_SIGNALING | 0.6849 |
| 90 | REACTOME_DNA_REPLICATION | 0.6836 |
| 91 | REACTOME_DNA_REPLICATION_PRE_INITIATION | 0.6830 |
| 92 | WP_EUKARYOTIC_TRANSCRIPTION_INITIATION | 0.6827 |
| 93 | REACTOME_DEGRADATION_OF_AXIN | 0.6824 |
| 94 | REACTOME_ANTIGEN_PRESENTATION_FOLDING_ASSEMBLY_AND_PEPTIDE_LOADING_OF_CLASS_I_MHC | 0.6805 |
| 95 | PID_BARD1_PATHWAY | 0.6799 |
| 96 | REACTOME_RECRUITMENT_OF_NUMA_TO_MITOTIC_CENTROSOMES | 0.6798 |
| 97 | WP_NUCLEOTIDE_EXCISION_REPAIR | 0.6787 |
| 98 | REACTOME_RESOLUTION_OF_AP_SITES_VIA_THE_MULTIPLE_NUCLEOTIDE_PATCH_REPLACEMENT_PATHWAY | 0.6769 |
| 99 | WP_MRNA_PROCESSING | 0.6757 |
| 100 | WP_DNA_REPAIR_PATHWAYS_FULL_NETWORK | 0.6750 |
| 101 | REACTOME_RESPIRATORY_ELECTRON_TRANSPORT | 0.6750 |
| 102 | REACTOME_HATS_ACETYLATE_HISTONES | 0.6738 |
| 103 | PID_AURORA_B_PATHWAY | 0.6738 |
| 104 | REACTOME_RESPONSE_OF_EIF2AK4_GCN2_TO_AMINO_ACID_DEFICIENCY | 0.6735 |
| 105 | WP_DNA_IR_DAMAGE_AND_CELLULAR_RESPONSE_VIA_ATR | 0.6727 |
| 106 | REACTOME_SARS_COV_2_MODULATES_HOST_TRANSLATION_MACHINERY | 0.6716 |
| 107 | REACTOME_RRNA_MODIFICATION_IN_THE_NUCLEUS_AND_CYTOSOL | 0.6708 |
| 108 | REACTOME_SYNTHESIS_OF_DNA | 0.6688 |
| 109 | WP_JOUBERT_SYNDROME | 0.6684 |
| 110 | KEGG_MEDICUS_VARIANT_MUTATION_CAUSED_ABERRANT_SOD1_TO_26S_PROTEASOME_MEDIATED_PROTEIN_DEGRADATION | 0.6674 |
| 111 | WP_INTEGRATED_CANCER_PATHWAY | 0.6673 |
| 112 | REACTOME_AURKA_ACTIVATION_BY_TPX2 | 0.6661 |
| 113 | REACTOME_APOPTOSIS | 0.6661 |
| 114 | WP_NUCLEOTIDE_EXCISION_REPAIR_IN_XERODERMA_PIGMENTOSUM | 0.6657 |
| 115 | REACTOME_ACTIVATION_OF_THE_PRE_REPLICATIVE_COMPLEX | 0.6632 |
| 116 | KEGG_MEDICUS_REFERENCE_MISMATCH_REPAIR | 0.6621 |
| 117 | REACTOME_RECOGNITION_OF_DNA_DAMAGE_BY_PCNA_CONTAINING_REPLICATION_COMPLEX | 0.6600 |
| 118 | REACTOME_MRNA_SPLICING_MINOR_PATHWAY | 0.6599 |
| 119 | REACTOME_OVARIAN_TUMOR_DOMAIN_PROTEASES | 0.6595 |
| 120 | REACTOME_NONSENSE_MEDIATED_DECAY_NMD | 0.6573 |
| 121 | WP_NAD_METABOLISM_IN_ONCOGENE_INDUCED_SENESCENCE_AND_MITOCHONDRIAL_DYSFUNCTION_ASSOCIATED_SENESCENCE | 0.6562 |
| 122 | REACTOME_HDR_THROUGH_HOMOLOGOUS_RECOMBINATION_HRR | 0.6559 |
| 123 | REACTOME_DNA_DAMAGE_BYPASS | 0.6553 |
| 124 | BIOCARTA_ATM_PATHWAY | 0.6460 |
| 125 | REACTOME_RESOLUTION_OF_D_LOOP_STRUCTURES_THROUGH_SYNTHESIS_DEPENDENT_STRAND_ANNEALING_SDSA | 0.6449 |
| 126 | REACTOME_OXIDATIVE_STRESS_INDUCED_SENESCENCE | 0.6437 |

Supplementary Table 9. Pathway ranking of LUAD copy number variation

| Rank | Pathway | Score (ARI) |
| --- | --- | --- |
| 1 | WP_B_CELL_RECEPTOR_SIGNALING_PATHWAY | 0.7356 |
| 2 | REACTOME_DNA_DOUBLE_STRAND_BREAK_REPAIR | 0.7157 |
| 3 | WP_NONALCOHOLIC_FATTY_LIVER_DISEASE | 0.7148 |
| 4 | REACTOME_SARS_COV_1_INFECTION | 0.7142 |
| 5 | KEGG_CALCIUM_SIGNALING_PATHWAY | 0.7075 |
| 6 | REACTOME_NUCLEOTIDE_EXCISION_REPAIR | 0.7067 |
| 7 | REACTOME_PROTEIN_LOCALIZATION | 0.7037 |
| 8 | WP_FRAGILE_X_SYNDROME | 0.7015 |
| 9 | REACTOME_EPIGENETIC_REGULATION_OF_GENE_EXPRESSION | 0.6963 |
| 10 | WP_DNA_REPAIR_PATHWAYS_FULL_NETWORK | 0.6930 |
| 11 | WP_CILIOPATHIES | 0.6895 |
| 12 | WP_MRNA_PROCESSING | 0.6800 |
| 13 | REACTOME_RESPIRATORY_ELECTRON_TRANSPORT_ATP_SYNTHESIS_BY_CHEMIOSMOTIC_COUPLING_AND_HEAT_PRODUCTION_BY_UNCOUPLING_PROTEINS | 0.6758 |
| 14 | KEGG_LYSOSOME | 0.6744 |
| 15 | WP_EGF_EGFR_SIGNALING_PATHWAY | 0.6675 |
| 16 | REACTOME_RESPIRATORY_ELECTRON_TRANSPORT | 0.6618 |
| 17 | WP_CELL_CYCLE | 0.6607 |
| 18 | REACTOME_THE_CITRIC_ACID_TCA_CYCLE_AND_RESPIRATORY_ELECTRON_TRANSPORT | 0.6541 |
| 19 | WP_CORTICOTROPIN_RELEASING_HORMONE_SIGNALING_PATHWAY | 0.6541 |
| 20 | REACTOME_METABOLISM_OF_STEROIDS | 0.6528 |
| 21 | KEGG_CELL_CYCLE | 0.6503 |
| 22 | KEGG_ADIPOCYTOKINE_SIGNALING_PATHWAY | 0.6467 |
| 23 | REACTOME_PEROXISOMAL_PROTEIN_IMPORT | 0.6438 |
| 24 | REACTOME_GLOBAL_GENOME_NUCLEOTIDE_EXCISION_REPAIR_GG_NER | 0.6437 |
| 25 | REACTOME_TRANSCRIPTION_COUPLED_NUCLEOTIDE_EXCISION_REPAIR_TC_NER | 0.6393 |
| 26 | PID_ERBB1_DOWNSTREAM_PATHWAY | 0.6363 |
| 27 | REACTOME_SIGNALING_BY_FGFR | 0.6357 |
| 28 | PID_P53_DOWNSTREAM_PATHWAY | 0.6347 |
| 29 | PID_PDGFRB_PATHWAY | 0.6341 |
| 30 | WP_ANDROGEN_RECEPTOR_NETWORK_IN_PROSTATE_CANCER | 0.6325 |
| 31 | REACTOME_HOMOLOGY_DIRECTED_REPAIR | 0.6325 |
| 32 | REACTOME_ESTROGEN_DEPENDENT_GENE_EXPRESSION | 0.6316 |
| 33 | WP_ERBB_SIGNALING_PATHWAY | 0.6314 |
| 34 | REACTOME_OPIOID_SIGNALLING | 0.6309 |
| 35 | WP_SUDDEN_INFANT_DEATH_SYNDROME_SIDS_SUSCEPTIBILITY_PATHWAYS | 0.6294 |
| 36 | WP_ENDODERM_DIFFERENTIATION | 0.6282 |
| 37 | REACTOME_CYTOSOLIC_SENSORS_OF_PATHOGEN_ASSOCIATED_DNA | 0.6275 |
| 38 | REACTOME_CELLULAR_SENESCENCE | 0.6247 |
| 39 | REACTOME_FCGAMMA_RECEPTOR_FCGR_DEPENDENT_PHAGOCYTOSIS | 0.6241 |
| 40 | PID_TCR_PATHWAY | 0.6237 |
| 41 | REACTOME_POTENTIAL_THERAPEUTICS_FOR_SARS | 0.6232 |
| 42 | KEGG_PROSTATE_CANCER | 0.6211 |
| 43 | KEGG_PHOSPHATIDYLINOSITOL_SIGNALING_SYSTEM | 0.6203 |
| 44 | WP_THYROID_HORMONES_PRODUCTION_AND_PERIPHERAL_DOWNSTREAM_SIGNALING_EFFECTS | 0.6188 |
| 45 | REACTOME_RNA_POLYMERASE_II_TRANSCRIBES_SNRNA_GENES | 0.6177 |
| 46 | WP_P53_TRANSCRIPTIONAL_GENE_NETWORK | 0.6160 |
| 47 | KEGG_PEROXISOME | 0.6147 |
| 48 | PID_FAK_PATHWAY | 0.6144 |
| 49 | WP_WNT_SIGNALING_PATHWAY | 0.6140 |
| 50 | KEGG_FC_GAMMA_R_MEDIATED_PHAGOCYTOSIS | 0.6140 |
| 51 | REACTOME_REGULATION_OF_TP53_ACTIVITY | 0.6125 |
| 52 | KEGG_ERBB_SIGNALING_PATHWAY | 0.6118 |
| 53 | WP_REGULATION_OF_ACTIN_CYTOSKELETON | 0.6116 |
| 54 | WP_NEURAL_CREST_DIFFERENTIATION | 0.6115 |
| 55 | PID_FCER1_PATHWAY | 0.6110 |
| 56 | WP_BARDET_BIEDL_SYNDROME | 0.6095 |
| 57 | WP_IL_18_SIGNALING_PATHWAY | 0.6095 |
| 58 | REACTOME_SIGNALING_BY_INSULIN_RECEPTOR | 0.6090 |
| 59 | REACTOME_EPH_EPHRIN_SIGNALING | 0.6083 |
| 60 | REACTOME_NUCLEAR_EVENTS_KINASE_AND_TRANSCRIPTION_FACTOR_ACTIVATION | 0.6078 |
| 61 | WP_BURN_WOUND_HEALING | 0.6077 |
| 62 | WP_OREXIN_RECEPTOR_PATHWAY | 0.6073 |
| 63 | REACTOME_GLUCOSE_METABOLISM | 0.6071 |
| 64 | WP_RANKL_RANK_SIGNALING_PATHWAY | 0.6064 |
| 65 | WP_PANCREATIC_ADENOCARCINOMA_PATHWAY | 0.6062 |
| 66 | WP_T_CELL_RECEPTOR_SIGNALING_PATHWAY | 0.6061 |
| 67 | WP_BRAIN_DERIVED_NEUROTROPHIC_FACTOR_BDNF_SIGNALING_PATHWAY | 0.6058 |
| 68 | WP_THYMIC_STROMAL_LYMPHOPOIETIN_TSLP_SIGNALING_PATHWAY | 0.6055 |
| 69 | KEGG_MEDICUS_REFERENCE_MITOCHONDRIAL_COMPLEX_UCP1_IN_THERMOGENESIS | 0.6050 |
| 70 | REACTOME_DNA_DOUBLE_STRAND_BREAK_RESPONSE | 0.6043 |
| 71 | WP_MELANOMA | 0.6041 |
| 72 | WP_UROTENSIN_II_MEDIATED_SIGNALING_PATHWAY | 0.6030 |
| 73 | KEGG_PARKINSONS_DISEASE | 0.6027 |
| 74 | REACTOME_COMPLEX_I_BIOGENESIS | 0.6020 |
| 75 | WP_MICRORNAS_IN_CARDIOMYOCYTE_HYPERTROPHY | 0.6008 |
| 76 | REACTOME_SIGNALING_BY_NOTCH1_PEST_DOMAIN_MUTANTS_IN_CANCER | 0.6000 |
| 77 | WP_CHROMOSOMAL_AND_MICROSATELLITE_INSTABILITY_IN_COLORECTAL_CANCER | 0.5995 |
| 78 | WP_TGF_BETA_SIGNALING_PATHWAY | 0.5985 |
| 79 | PID_FOXO_PATHWAY | 0.5977 |
| 80 | REACTOME_ISG15_ANTIVIRAL_MECHANISM | 0.5971 |
| 81 | REACTOME_TP53_REGULATES_METABOLIC_GENES | 0.5956 |
| 82 | REACTOME_HIV_LIFE_CYCLE | 0.5948 |
| 83 | REACTOME_MITOCHONDRIAL_TRANSLATION | 0.5941 |
| 84 | PID_P53_REGULATION_PATHWAY | 0.5937 |
| 85 | REACTOME_TRANS_GOLGI_NETWORK_VESICLE_BUDDING | 0.5937 |
| 86 | REACTOME_DNA_DAMAGE_BYPASS | 0.5936 |
| 87 | PID_MYC_ACTIV_PATHWAY | 0.5915 |
| 88 | WP_DNA_IR_DAMAGE_AND_CELLULAR_RESPONSE_VIA_ATR | 0.5911 |
| 89 | REACTOME_GLYCOLYSIS | 0.5911 |
| 90 | REACTOME_INTRINSIC_PATHWAY_FOR_APOPTOSIS | 0.5910 |
| 91 | PID_AP1_PATHWAY | 0.5897 |
| 92 | WP_EGFR_TYROSINE_KINASE_INHIBITOR_RESISTANCE | 0.5891 |
| 93 | REACTOME_SIGNALING_BY_TGFB_FAMILY_MEMBERS | 0.5868 |
| 94 | WP_MITOCHONDRIAL_COMPLEX_I_ASSEMBLY_MODEL_OXPHOS_SYSTEM | 0.5855 |
| 95 | SIG_INSULIN_RECEPTOR_PATHWAY_IN_CARDIAC_MYOCYTES | 0.5837 |
| 96 | REACTOME_SARS_COV_1_HOST_INTERACTIONS | 0.5821 |
| 97 | WP_LEPTIN_SIGNALING_PATHWAY | 0.5814 |
| 98 | REACTOME_RNA_POLYMERASE_II_PRE_TRANSCRIPTION_EVENTS | 0.5798 |
| 99 | REACTOME_RHOA_GTPASE_CYCLE | 0.5795 |
| 100 | REACTOME_MRNA_SPLICING_MINOR_PATHWAY | 0.5790 |
| 101 | REACTOME_SIGNALING_BY_FGFR2 | 0.5778 |
| 102 | KEGG_CHRONIC_MYELOID_LEUKEMIA | 0.5768 |
| 103 | KEGG_MEDICUS_REFERENCE_GF_RTK_PI3K_SIGNALING_PATHWAY | 0.5722 |
| 104 | REACTOME_HDR_THROUGH_HOMOLOGOUS_RECOMBINATION_HRR | 0.5687 |
| 105 | REACTOME_RAC2_GTPASE_CYCLE | 0.5686 |

Supplementary Table 10. Evidence of cancer-related miRNAs selected by IPFMC’s association with cancer

| miRNA | supporting literature |
| --- | --- |
| hsa-mir-199a (-1, -2) | [4] |
| hsa-mir-429 | [5] |
| hsa-mir-34a | [6] |
| hsa-mir-155 | [7] |
| hsa-mir-27a | [8] |
| hsa-mir-200c | [9] |
| hsa-mir-203a | - |
| hsa-mir-451a | - |
| hsa-mir-145 | [10] |
| hsa-mir-9 (-1,-2,-3) | [11] |
| hsa-mir-125a | [12] |
| hsa-mir-200b | [13] |
| hsa-mir-497 | [14] |
| hsa-mir-181a-1 | [15] |
| hsa-mir-126 | [16] |
| hsa-mir-139 | [17] |
| hsa-mir-196b | [18] |

Supplementary Table 11. Top 100 genes/miRNAs of LUAD’s four omics

| Rank | mRNA | | miRNA | | Methylation | | CNV | |
| --- | --- | --- | --- | --- | --- | --- | --- | --- |
|  | Gene | Count | miRNA | Count | Gene | Count | Gene | Count |
| 1 | GRB2 | 88 | hsa-mir-199a-1 | 127 | RPS27A | 57 | MAPK1 | 41 |
| 2 | MAPK1 | 88 | hsa-mir-199a-2 | 127 | UBA52 | 55 | AKT1 | 36 |
| 3 | SOS1 | 87 | hsa-mir-429 | 121 | UBB | 54 | MAPK3 | 34 |
| 4 | HRAS | 87 | hsa-mir-34a | 121 | UBC | 53 | PIK3R1 | 33 |
| 5 | MAPK3 | 85 | hsa-mir-155 | 121 | PSMA3 | 50 | MAPK8 | 30 |
| 6 | PIK3R1 | 78 | hsa-mir-27a | 119 | PSMC1 | 49 | MAP2K1 | 28 |
| 7 | MAP2K1 | 77 | hsa-mir-200c | 119 | PSMC3 | 49 | PIK3R2 | 27 |
| 8 | PIK3CA | 77 | hsa-mir-203a | 118 | PSMC4 | 49 | GRB2 | 26 |
| 9 | RAF1 | 72 | hsa-mir-451a | 118 | PSMC5 | 49 | PIK3CA | 26 |
| 10 | KRAS | 70 | hsa-mir-145 | 117 | PSMC6 | 49 | RAF1 | 26 |
| 11 | AKT1 | 64 | hsa-mir-9-2 | 117 | PSMD1 | 49 | SOS1 | 25 |
| 12 | NRAS | 63 | hsa-mir-9-3 | 117 | PSMD11 | 49 | MAPK9 | 24 |
| 13 | MAP2K2 | 61 | hsa-mir-9-1 | 117 | PSMD12 | 49 | TP53 | 24 |
| 14 | BRAF | 51 | hsa-mir-125a | 115 | PSMD13 | 49 | NFKB1 | 23 |
| 15 | SHC1 | 49 | hsa-mir-200b | 115 | PSMD2 | 49 | EP300 | 22 |
| 16 | PIK3R2 | 49 | hsa-mir-497 | 114 | PSMD3 | 49 | UBB | 22 |
| 17 | SOS2 | 47 | hsa-mir-181a-1 | 113 | PSMD4 | 49 | MAP2K2 | 22 |
| 18 | MAPK8 | 45 | hsa-mir-126 | 113 | PSMD6 | 49 | GSK3B | 22 |
| 19 | SRC | 45 | hsa-mir-139 | 111 | PSMD8 | 49 | MTOR | 21 |
| 20 | PIK3CB | 43 | hsa-mir-196b | 111 | PSMC2 | 49 | RPS27A | 21 |
| 21 | AKT2 | 41 | hsa-mir-143 | 111 | PSMA1 | 49 | PLCG1 | 21 |
| 22 | PIK3CD | 41 | hsa-mir-148a | 108 | PSMA4 | 49 | UBC | 21 |
| 23 | RAC1 | 40 | hsa-let-7a-1 | 106 | PSMB1 | 49 | HRAS | 21 |
| 24 | JUN | 38 | hsa-mir-21 | 105 | PSMA5 | 49 | UBA52 | 21 |
| 25 | PLCG1 | 37 | hsa-mir-23b | 104 | PSMA2 | 49 | JUN | 20 |
| 26 | ARAF | 37 | hsa-mir-19a | 103 | PSMB3 | 49 | RELA | 19 |
| 27 | PIK3R3 | 36 | hsa-mir-15b | 102 | PSMB4 | 49 | RAC1 | 19 |
| 28 | AKT3 | 35 | hsa-mir-365a | 102 | PSMA6 | 49 | AKT2 | 19 |
| 29 | BAD | 34 | hsa-let-7a-2 | 102 | PSMB5 | 49 | MAPK14 | 18 |
| 30 | PIK3CG | 34 | hsa-mir-30a | 102 | PSMB6 | 49 | SHC1 | 18 |
| 31 | GAB1 | 33 | hsa-mir-221 | 101 | PSMB7 | 49 | PIK3CB | 18 |
| 32 | MAPK14 | 32 | hsa-let-7a-3 | 100 | PSMA7 | 49 | SRC | 18 |
| 33 | EGFR | 32 | hsa-mir-96 | 99 | PSMD7 | 49 | BRAF | 18 |
| 34 | CDC42 | 32 | hsa-let-7c | 97 | PSMB2 | 49 | PTPN11 | 18 |
| 35 | PTPN11 | 31 | hsa-mir-195 | 97 | PSME3 | 48 | PTEN | 17 |
| 36 | GSK3B | 31 | hsa-mir-29a | 97 | PSME2 | 48 | AKT3 | 17 |
| 37 | RHOA | 30 | hsa-mir-7-3 | 96 | PSME1 | 48 | KRAS | 17 |
| 38 | STAT3 | 30 | hsa-mir-101-2 | 96 | PSMD9 | 48 | CDC42 | 17 |
| 39 | MTOR | 30 | hsa-mir-375 | 96 | PSMB9 | 48 | MYC | 17 |
| 40 | ELK1 | 29 | hsa-mir-101-1 | 96 | PSMB8 | 48 | IKBKB | 17 |
| 41 | NFKB1 | 29 | hsa-mir-193a | 94 | PSMD14 | 48 | SP1 | 17 |
| 42 | MAPK9 | 28 | hsa-mir-486-2 | 94 | PSMB10 | 48 | PDPK1 | 16 |
| 43 | PAK1 | 28 | hsa-mir-486-1 | 94 | PSMD10 | 47 | CHUK | 16 |
| 44 | FOS | 27 | hsa-mir-146a | 94 | PSMF1 | 47 | PRKCA | 16 |
| 45 | PDPK1 | 27 | hsa-mir-17 | 94 | PSMD5 | 47 | PIK3CD | 16 |
| 46 | CBL | 26 | hsa-mir-184 | 94 | PSMA8 | 40 | PIK3R3 | 16 |
| 47 | EGF | 26 | hsa-mir-214 | 94 | PSMB11 | 39 | CCND1 | 15 |
| 48 | PRKCA | 26 | hsa-mir-224 | 90 | PSME4 | 39 | PCNA | 15 |
| 49 | IKBKB | 25 | hsa-mir-22 | 90 | SEH1L | 34 | STAT3 | 15 |
| 50 | CCND1 | 25 | hsa-mir-200a | 90 | SEC13 | 34 | CTNNB1 | 15 |
| 51 | MAP2K4 | 24 | hsa-mir-708 | 89 | NUP85 | 34 | CREB1 | 15 |
| 52 | PTEN | 24 | hsa-mir-15a | 89 | NUP43 | 34 | PRKCB | 15 |
| 53 | RPS6KB1 | 24 | hsa-mir-542 | 88 | NUP37 | 34 | FOS | 15 |
| 54 | CRK | 23 | hsa-mir-34c | 88 | RANBP2 | 34 | CBL | 14 |
| 55 | CREB1 | 23 | hsa-mir-217 | 88 | NUP107 | 34 | PAK1 | 14 |
| 56 | CHUK | 22 | hsa-mir-182 | 88 | NUP133 | 34 | RHOA | 14 |
| 57 | RELA | 22 | hsa-mir-543 | 86 | NUP160 | 34 | BAD | 14 |
| 58 | TP53 | 21 | hsa-mir-29c | 86 | NUP98 | 33 | PRKCD | 14 |
| 59 | PIK3R5 | 21 | hsa-let-7b | 84 | NUP214 | 31 | IKBKG | 14 |
| 60 | IRS1 | 20 | hsa-mir-133a-1 | 84 | NDC1 | 30 | SMAD3 | 14 |
| 61 | STAT1 | 20 | hsa-mir-133a-2 | 84 | POM121 | 30 | BAX | 13 |
| 62 | MYC | 20 | hsa-mir-132 | 83 | NUP93 | 30 | CDKN1A | 13 |
| 63 | PTK2 | 20 | hsa-mir-93 | 82 | NUP88 | 30 | NFKBIA | 13 |
| 64 | ERBB2 | 19 | hsa-mir-98 | 82 | NUP155 | 30 | TGFB1 | 13 |
| 65 | IKBKG | 19 | hsa-mir-204 | 82 | NUP62 | 30 | NRAS | 13 |
| 66 | NFKBIA | 19 | hsa-mir-320a | 81 | NUP54 | 30 | CDK2 | 13 |
| 67 | CDKN1A | 19 | hsa-mir-223 | 80 | NUP188 | 30 | MDM2 | 13 |
| 68 | PRKCB | 19 | hsa-mir-449a | 80 | NUP35 | 30 | CREBBP | 13 |
| 69 | MAP3K1 | 18 | hsa-mir-34b | 79 | NUP210 | 30 | RPS6KB1 | 13 |
| 70 | PAK2 | 18 | hsa-mir-491 | 79 | NUP205 | 30 | STAT1 | 12 |
| 71 | MAPK10 | 18 | hsa-mir-20a | 78 | NUP50 | 29 | PTK2 | 12 |
| 72 | PRKCD | 18 | hsa-mir-28 | 77 | POM121C | 29 | GAB1 | 12 |
| 73 | FOXO3 | 17 | hsa-mir-142 | 76 | TPR | 29 | ELK1 | 12 |
| 74 | MAP2K7 | 17 | hsa-mir-330 | 74 | AAAS | 29 | HDAC1 | 12 |
| 75 | EIF4EBP1 | 17 | hsa-mir-503 | 74 | NUP153 | 29 | CASP3 | 12 |
| 76 | MAP2K3 | 17 | hsa-mir-424 | 74 | RAE1 | 29 | ATM | 12 |
| 77 | MAP2K6 | 17 | hsa-mir-23a | 73 | RBX1 | 22 | SFN | 12 |
| 78 | SHC2 | 16 | hsa-mir-106a | 73 | SKP1 | 20 | POLK | 12 |
| 79 | FOXO1 | 16 | hsa-mir-33b | 72 | CUL1 | 19 | EGFR | 12 |
| 80 | CTNNB1 | 16 | hsa-mir-100 | 72 | RPA1 | 19 | FYN | 12 |
| 81 | STAT5A | 16 | hsa-mir-185 | 71 | RPA2 | 18 | E2F1 | 12 |
| 82 | RPS27A | 15 | hsa-mir-433 | 71 | CDK2 | 18 | CRK | 12 |
| 83 | UBC | 15 | hsa-mir-26a-1 | 70 | RPA3 | 17 | CDKN2A | 12 |
| 84 | UBB | 15 | hsa-mir-362 | 69 | CDK1 | 17 | ABL1 | 12 |
| 85 | MAPK11 | 15 | hsa-mir-425 | 69 | PCNA | 17 | VAV1 | 11 |
| 86 | RPS6KA1 | 15 | hsa-mir-181b-1 | 69 | POLE | 15 | SMAD4 | 11 |
| 87 | CRKL | 15 | hsa-mir-141 | 68 | POLE2 | 15 | YWHAH | 11 |
| 88 | MAP3K7 | 15 | hsa-mir-106b | 67 | POLE3 | 15 | YWHAG | 11 |
| 89 | STAT5B | 15 | hsa-mir-140 | 66 | POLE4 | 15 | YWHAB | 11 |
| 90 | UBA52 | 15 | hsa-mir-326 | 65 | TP53 | 15 | YWHAE | 11 |
| 91 | NCK1 | 14 | hsa-mir-152 | 65 | RFC4 | 14 | EGF | 11 |
| 92 | ATF2 | 14 | hsa-mir-27b | 64 | RFC3 | 14 | CDK7 | 11 |
| 93 | TGFA | 14 | hsa-mir-20b | 63 | RFC2 | 14 | POLR2F | 11 |
| 94 | MET | 14 | hsa-mir-296 | 62 | RFC5 | 14 | PPP2CA | 11 |
| 95 | PRKCZ | 14 | hsa-mir-144 | 62 | PLK1 | 13 | POLR2E | 11 |
| 96 | TGFB1 | 14 | hsa-mir-205 | 62 | SKP2 | 13 | YWHAQ | 11 |
| 97 | CASP3 | 14 | hsa-mir-194-1 | 62 | UBE2D1 | 13 | POLR2L | 11 |
| 98 | SHC3 | 14 | hsa-mir-194-2 | 62 | RFC1 | 12 | PLCG2 | 11 |
| 99 | RAPGEF1 | 14 | hsa-mir-10b | 61 | ATM | 12 | NCK1 | 11 |
| 100 | RAP1A | 14 | hsa-mir-370 | 61 | POLD1 | 12 | POLR2K | 11 |

Supplementary Table 12. Ablation experiment results (log-rank p-value) of IPFMC (NPS)

| Cancer | Combination | Number of clusters | | | | | | |
| --- | --- | --- | --- | --- | --- | --- | --- | --- |
|  |  | 2 | 3 | 4 | 5 | 6 | 7 | 8 |
| BRCA | m_mi | 0.1518 | 0.0224 | 0.0456 | 0.0734 | 0.1369 | 0.1456 | 0.5236 |
|  | m_me | 0.0242 | 0.0148 | 0.3684 | 0.1164 | 0.1391 | 0.1471 | 0.1897 |
|  | m_cnv | 0.0068 | 0.0466 | 0.0251 | 0.0126 | 0.0188 | 0.0303 | 0.0254 |
|  | mi_me | 0.2705 | 0.3992 | 0.5628 | 0.6975 | 0.6147 | 0.8299 | 0.5572 |
|  | mi_cnv | 0.0499 | 0.0162 | 0.0739 | 0.0434 | 0.1432 | 0.1532 | 0.1386 |
|  | me_cnv | 0.0138 | 0.1294 | 0.0188 | 0.1134 | 0.0547 | 0.0590 | 0.1261 |
|  | m_mi_me | 0.1112 | 0.0329 | 0.0684 | 0.0895 | 0.1155 | 0.9060 | 0.6220 |
|  | m_mi_cnv | 0.0410 | 0.0896 | 0.0218 | 0.0065 | 0.0260 | 0.0113 | 0.1138 |
|  | m_me_cnv | 0.0024 | 0.0516 | 0.0037 | 0.0041 | 0.0311 | 0.0048 | 0.0127 |
|  | mi_me_cnv | 0.0367 | 0.1760 | 0.3615 | 0.0330 | 0.2297 | 0.3774 | 0.3524 |
|  | m_mi_me_cnv | 0.0219 | 0.1670 | 0.1068 | 0.0029 | 0.0753 | 0.0776 | 0.0242 |
| COAD | m_mi | 0.5882 | 0.2336 | 0.1108 | 0.0739 | 0.2141 | 0.1267 | 0.0209 |
|  | m_me | 0.0935 | 0.0071 | 0.0092 | 0.0034 | 0.2941 | 0.1991 | 0.3451 |
|  | m_cnv | 0.6191 | 0.0627 | 0.0034 | 0.0102 | 0.0103 | 0.0803 | 0.0691 |
|  | mi_me | 0.7680 | 0.3555 | 0.2718 | 0.0627 | 0.2328 | 0.5946 | 0.0710 |
|  | mi_cnv | 0.9872 | 0.2764 | 0.0459 | 0.0105 | 0.0258 | 0.1067 | 0.0935 |
|  | me_cnv | 0.8049 | 0.2012 | 0.4169 | 0.0429 | 0.0440 | 0.0223 | 0.0863 |
|  | m_mi_me | 0.6097 | 0.1239 | 0.1941 | 0.4245 | 0.2203 | 0.2202 | 0.0669 |
|  | m_mi_cnv | 0.9259 | 0.7217 | 0.4411 | 0.5247 | 0.0813 | 0.0742 | 0.0341 |
|  | m_me_cnv | 0.8967 | 0.1480 | 0.0597 | 0.0227 | 0.0155 | 0.0223 | 0.0215 |
|  | mi_me_cnv | 0.5822 | 0.7519 | 0.1548 | 0.0851 | 0.1189 | 0.1093 | 0.1495 |
|  | m_mi_me_cnv | 0.6065 | 0.4032 | 0.4585 | 0.3698 | 0.1702 | 0.0638 | 0.0325 |
| KIRC | m_mi | 0.0000 | 0.0000 | 0.0002 | 0.0025 | 0.0005 | 0.0003 | 0.0000 |
|  | m_me | 0.0001 | 0.0000 | 0.0000 | 0.0000 | 0.0000 | 0.0003 | 0.0000 |
|  | m_cnv | 0.0638 | 0.0098 | 0.0167 | 0.0238 | 0.0059 | 0.0202 | 0.0111 |
|  | mi_me | 0.0006 | 0.0547 | 0.0000 | 0.0000 | 0.0000 | 0.0000 | 0.0000 |
|  | mi_cnv | 0.1247 | 0.0132 | 0.0008 | 0.0001 | 0.0041 | 0.0000 | 0.0002 |
|  | me_cnv | 0.2974 | 0.0258 | 0.0018 | 0.0001 | 0.0003 | 0.0006 | 0.0004 |
|  | m_mi_me | 0.0000 | 0.0008 | 0.0000 | 0.0000 | 0.0003 | 0.0014 | 0.0004 |
|  | m_mi_cnv | 0.0065 | 0.0025 | 0.0012 | 0.0001 | 0.0007 | 0.0001 | 0.0026 |
|  | m_me_cnv | 0.0858 | 0.0012 | 0.0084 | 0.0071 | 0.0002 | 0.0000 | 0.0000 |
|  | mi_me_cnv | 0.1037 | 0.0128 | 0.0028 | 0.0016 | 0.0000 | 0.0000 | 0.0000 |
|  | m_mi_me_cnv | 0.0070 | 0.0094 | 0.0001 | 0.0014 | 0.0000 | 0.0001 | 0.0000 |
| LUAD | m_mi | 0.1954 | 0.0413 | 0.0058 | 0.0002 | 0.0014 | 0.0005 | 0.0008 |
|  | m_me | 0.4764 | 0.0966 | 0.1980 | 0.1852 | 0.1534 | 0.0529 | 0.1540 |
|  | m_cnv | 0.2359 | 0.3641 | 0.3736 | 0.2745 | 0.2282 | 0.2872 | 0.1065 |
|  | mi_me | 0.0818 | 0.1268 | 0.1760 | 0.5584 | 0.6200 | 0.1003 | 0.0738 |
|  | mi_cnv | 0.0938 | 0.6232 | 0.0907 | 0.1297 | 0.2959 | 0.4832 | 0.1433 |
|  | me_cnv | 0.0860 | 0.2077 | 0.4097 | 0.3207 | 0.3791 | 0.5355 | 0.6970 |
|  | m_mi_me | 0.2826 | 0.0114 | 0.0048 | 0.0461 | 0.0414 | 0.2208 | 0.0086 |
|  | m_mi_cnv | 0.0628 | 0.5773 | 0.0241 | 0.0123 | 0.0092 | 0.0326 | 0.0194 |
|  | m_me_cnv | 0.0374 | 0.1195 | 0.2271 | 0.5482 | 0.3169 | 0.6438 | 0.3323 |
|  | mi_me_cnv | 0.0669 | 0.2184 | 0.3323 | 0.2806 | 0.3698 | 0.1067 | 0.2440 |
|  | m_mi_me_cnv | 0.1602 | 0.4252 | 0.0528 | 0.0399 | 0.2262 | 0.0044 | 0.0001 |
| LUSC | m_mi | 0.3890 | 0.1112 | 0.0494 | 0.1160 | 0.1855 | 0.1903 | 0.0492 |
|  | m_me | 0.8615 | 0.7937 | 0.7005 | 0.2632 | 0.0269 | 0.0323 | 0.0992 |
|  | m_cnv | 0.4336 | 0.8205 | 0.4114 | 0.7521 | 0.8310 | 0.8650 | 0.8598 |
|  | mi_me | 0.5063 | 0.0208 | 0.0206 | 0.0367 | 0.0415 | 0.1581 | 0.0968 |
|  | mi_cnv | 0.4575 | 0.2533 | 0.4332 | 0.2711 | 0.3662 | 0.4388 | 0.5022 |
|  | me_cnv | 0.9414 | 0.6103 | 0.4511 | 0.4720 | 0.4173 | 0.7107 | 0.7723 |
|  | m_mi_me | 0.4271 | 0.1343 | 0.0800 | 0.0686 | 0.0899 | 0.0118 | 0.0131 |
|  | m_mi_cnv | 0.4926 | 0.2746 | 0.3751 | 0.2687 | 0.2443 | 0.1676 | 0.4018 |
|  | m_me_cnv | 0.3961 | 0.7610 | 0.8506 | 0.3578 | 0.6513 | 0.4577 | 0.7350 |
|  | mi_me_cnv | 0.9510 | 0.3255 | 0.3763 | 0.2648 | 0.0363 | 0.1328 | 0.1262 |
|  | m_mi_me_cnv | 0.8932 | 0.0813 | 0.3696 | 0.3665 | 0.3976 | 0.0392 | 0.1182 |
| ACC | m_mi | 0.0000 | 0.0008 | 0.0009 | 0.0013 | 0.0060 | 0.0110 | 0.0001 |
|  | m_me | 0.0000 | 0.0000 | 0.0002 | 0.0000 | 0.0001 | 0.0002 | 0.0000 |
|  | m_cnv | 0.0021 | 0.0070 | 0.0065 | 0.0249 | 0.0078 | 0.0012 | 0.0166 |
|  | mi_me | 0.0002 | 0.0000 | 0.0000 | 0.0000 | 0.0022 | 0.0033 | 0.0001 |
|  | mi_cnv | 0.0004 | 0.0024 | 0.0014 | 0.0091 | 0.0280 | 0.0408 | 0.0001 |
|  | me_cnv | 0.0000 | 0.0011 | 0.0067 | 0.0312 | 0.1074 | 0.0057 | 0.0044 |
|  | m_mi_me | 0.0000 | 0.0000 | 0.0000 | 0.0000 | 0.0001 | 0.0076 | 0.0090 |
|  | m_mi_cnv | 0.0000 | 0.0008 | 0.0042 | 0.0016 | 0.0049 | 0.0027 | 0.0236 |
|  | m_me_cnv | 0.0000 | 0.0002 | 0.0030 | 0.0096 | 0.0000 | 0.0010 | 0.0002 |
|  | mi_me_cnv | 0.0004 | 0.0018 | 0.0004 | 0.0017 | 0.0076 | 0.0186 | 0.1051 |
|  | m_mi_me_cnv | 0.0000 | 0.0000 | 0.0000 | 0.0000 | 0.0008 | 0.0000 | 0.0049 |
| KIRP | m_mi | 0.1109 | 0.5059 | 0.2269 | 0.2030 | 0.5730 | 0.1082 | 0.4065 |
|  | m_me | 0.6382 | 0.4749 | 0.1870 | 0.1164 | 0.0559 | 0.1405 | 0.0000 |
|  | m_cnv | 0.4787 | 0.1570 | 0.0005 | 0.0069 | 0.0121 | 0.0154 | 0.0365 |
|  | mi_me | 0.2684 | 0.1515 | 0.0664 | 0.0169 | 0.0031 | 0.0033 | 0.0000 |
|  | mi_cnv | 0.5175 | 0.1731 | 0.0219 | 0.0038 | 0.0048 | 0.0006 | 0.0050 |
|  | me_cnv | 0.0012 | 0.0001 | 0.0002 | 0.0001 | 0.0001 | 0.0000 | 0.0000 |
|  | m_mi_me | 0.0812 | 0.2446 | 0.1874 | 0.3607 | 0.4306 | 0.0128 | 0.1228 |
|  | m_mi_cnv | 0.0648 | 0.2352 | 0.2900 | 0.1033 | 0.5054 | 0.4697 | 0.5136 |
|  | m_me_cnv | 0.0012 | 0.0000 | 0.0000 | 0.0000 | 0.0000 | 0.0000 | 0.0000 |
|  | mi_me_cnv | 0.0006 | 0.0000 | 0.0002 | 0.0000 | 0.0000 | 0.0000 | 0.0000 |
|  | m_mi_me_cnv | 0.0256 | 0.0708 | 0.0690 | 0.0000 | 0.0000 | 0.0000 | 0.0000 |
| LIHC | m_mi | 0.8212 | 0.7104 | 0.3134 | 0.6800 | 0.5062 | 0.4909 | 0.6842 |
|  | m_me | 0.7943 | 0.7318 | 0.8644 | 0.7194 | 0.8532 | 0.9483 | 0.7676 |
|  | m_cnv | 0.4138 | 0.6889 | 0.0216 | 0.0067 | 0.0152 | 0.0842 | 0.0729 |
|  | mi_me | 0.3078 | 0.5484 | 0.5957 | 0.7522 | 0.9210 | 0.9601 | 0.9890 |
|  | mi_cnv | 0.1958 | 0.5476 | 0.2380 | 0.9149 | 0.5544 | 0.5911 | 0.3977 |
|  | me_cnv | 0.3417 | 0.1290 | 0.4006 | 0.2953 | 0.5880 | 0.3836 | 0.5689 |
|  | m_mi_me | 0.7185 | 0.7063 | 0.6091 | 0.3482 | 0.5759 | 0.9593 | 0.8770 |
|  | m_mi_cnv | 0.8892 | 0.6341 | 0.5132 | 0.5236 | 0.7606 | 0.7397 | 0.3023 |
|  | m_me_cnv | 0.3296 | 0.3652 | 0.4573 | 0.7158 | 0.1228 | 0.0331 | 0.1497 |
|  | mi_me_cnv | 0.4163 | 0.0935 | 0.4468 | 0.4709 | 0.2319 | 0.7020 | 0.5983 |
|  | m_mi_me_cnv | 0.0937 | 0.1850 | 0.6249 | 0.7286 | 0.8156 | 0.8064 | 0.8589 |
| THYM | m_mi | 0.0041 | 0.0036 | 0.0217 | 0.0324 | 0.0046 | 0.0172 | 0.0260 |
|  | m_me | 0.0020 | 0.0146 | 0.0173 | 0.0306 | 0.0546 | 0.0730 | 0.0970 |
|  | m_cnv | 0.0002 | 0.0003 | 0.0007 | 0.0032 | 0.0017 | 0.0012 | 0.0047 |
|  | mi_me | 0.0033 | 0.0303 | 0.0006 | 0.0014 | 0.0034 | 0.0034 | 0.0061 |
|  | mi_cnv | 0.0000 | 0.0001 | 0.0000 | 0.0003 | 0.0017 | 0.0036 | 0.0035 |
|  | me_cnv | 0.0008 | 0.0109 | 0.0018 | 0.0046 | 0.0071 | 0.0145 | 0.0040 |
|  | m_mi_me | 0.0037 | 0.0117 | 0.0018 | 0.0134 | 0.0086 | 0.0116 | 0.0019 |
|  | m_mi_cnv | 0.0000 | 0.0001 | 0.0004 | 0.0000 | 0.0025 | 0.0039 | 0.0045 |
|  | m_me_cnv | 0.0026 | 0.0058 | 0.0119 | 0.0250 | 0.0233 | 0.0388 | 0.0526 |
|  | mi_me_cnv | 0.0000 | 0.0001 | 0.0009 | 0.0010 | 0.0018 | 0.0039 | 0.0057 |
|  | m_mi_me_cnv | 0.0003 | 0.0010 | 0.0011 | 0.0020 | 0.0047 | 0.0033 | 0.0044 |

IPFMC(NPS): IPFMC without iterative pathway selection step

**Supplementary References**

1. Hubert L, Arabie P. Comparing partitions, Journal of Classification 1985;2:193-218.

2. Strehl A, Ghosh J. Cluster Ensembles - A Knowledge Reuse Framework for Combining Multiple Partitions, Journal of Machine Learning Research 2002;3:583-617.

3. Duan R, Gao L, Gao Y et al. Evaluation and comparison of multi-omics data integration methods for cancer subtyping, PLoS Comput Biol 2021;17:e1009224.

4. Ahmadi A, Khansarinejad B, Hosseinkhani S et al. miR-199a-5p and miR-495 target GRP78 within UPR pathway of lung cancer, Gene 2017;620:15-22.

5. Zhang CW, Zhou B, Liu YC et al. LINC00365 inhibited lung adenocarcinoma progression and glycolysis via sponging miR-429/KCTD12 axis, Environ Toxicol 2022;37:1853-1866.

6. Wen Y, Huang H, Huang B et al. HSA-miR-34a-5p regulates the SIRT1/TP53 axis in prostate cancer, Am J Transl Res 2022;14:4493-4504.

7. Yao Q, Zhang AM, Ma H et al. Novel molecular beacons to monitor microRNAs in non-small-cell lung cancer, Mol Cell Probes 2012;26:182-187.

8. Wang Z, Lai J, Wang Y et al. The Hsa-miR-27a rs895819 (A>G) polymorphism and cancer susceptibility, Gene 2013;521:87-90.

9. Zhang DD, Li Y, Xu Y et al. Phosphodiesterase 7B/microRNA-200c relationship regulates triple-negative breast cancer cell growth, Oncogene 2019;38:1106-1120.

10. Cho WC, Chow AS, Au JS. Restoration of tumour suppressor hsa-miR-145 inhibits cancer cell growth in lung adenocarcinoma patients with epidermal growth factor receptor mutation, Eur J Cancer 2009;45:2197-2206.

11. Wu S, Xu J, Zhang M et al. Analysis of Genetic Variants and the ceRNA Network of miR-9 in Non-Small Cell Lung Cancer, DNA Cell Biol 2022;41:142-150.

12. Jiang L, Chang J, Zhang Q et al. MicroRNA hsa-miR-125a-3p activates p53 and induces apoptosis in lung cancer cells, Cancer Invest 2013;31:538-544.

13. Feng L, Cai W, Jin S et al. Integrated bioinformatics analysis of microarray data from non-small cell lung cancer, Cell Mol Biol (Noisy-le-grand) 2023;69:218-224.

14. Xia Y, Hu C, Lian L et al. miR‑497 suppresses malignant phenotype in non‑small cell lung cancer via targeting KDR, Oncol Rep 2019;42:443-452.

15. Chen Y, Liao W, Yuan A et al. MiR-181a reduces radiosensitivity of non-small-cell lung cancer via inhibiting PTEN, Panminerva Med 2022;64:374-383.

16. Zhu X, Li H, Long L et al. miR-126 enhances the sensitivity of non-small cell lung cancer cells to anticancer agents by targeting vascular endothelial growth factor A, Acta Biochim Biophys Sin (Shanghai) 2012;44:519-526.

17. Du H, Bao Y, Liu C et al. miR‑139‑5p enhances cisplatin sensitivity in non‑small cell lung cancer cells by inhibiting cell proliferation and promoting apoptosis via the targeting of Homeobox protein Hox‑B2, Mol Med Rep 2021;23.

18. Xiong L, He X, Wang L et al. Hypoxia-associated prognostic markers and competing endogenous RNA coexpression networks in lung adenocarcinoma, Sci Rep 2022;12:21340.
